# The engineered CD80 variant fusion therapeutic davoceticept combines checkpoint antagonism with conditional CD28 costimulation for anti-tumor immunity

**DOI:** 10.1038/s41467-022-29286-5

**Published:** 2022-04-04

**Authors:** Mark F. Maurer, Katherine E. Lewis, Joseph L. Kuijper, Dan Ardourel, Chelsea J. Gudgeon, Siddarth Chandrasekaran, Sherri L. Mudri, Kayla N. Kleist, Chris Navas, Martin F. Wolfson, Mark W. Rixon, Ryan Swanson, Stacey R. Dillon, Steven D. Levin, Yengo Raymond Kimbung, Masato Akutsu, Derek T. Logan, Björn Walse, Kristine M. Swiderek, Stanford L. Peng

**Affiliations:** 1 Alpine Immune Sciences, Inc., Seattle, WA USA; 2 SARomics Biostructures AB, Medicon Villiage, Lund, Sweden; 3 Present Address: Notch Therapeutics, Inc., Seattle, WA USA; 4 Present Address: Lyell Immunopharma, Inc., Seattle, WA USA; 5 Present Address: Neoleukin Therapeutics, Inc., Seattle, WA USA; 6 Present Address: Parvus Therapeutics, Inc., South San Francisco, CA USA; 7 Present Address: Department of Chemistry, Keio University, Yokohama, Japan; 8 Present Address: Mozart Therapeutics, Inc., Seattle, WA USA

**Keywords:** Recombinant protein therapy, Cancer immunotherapy, Recombinant protein therapy

## Abstract

Despite the recent clinical success of T cell checkpoint inhibition targeting the CTLA-4 and PD-1 pathways, many patients either fail to achieve objective responses or they develop resistance to therapy. In some cases, poor responses to checkpoint blockade have been linked to suboptimal CD28 costimulation and the inability to generate and maintain a productive adaptive anti-tumor immune response. To address this, here we utilize directed evolution to engineer a CD80 IgV domain with increased PD-L1 affinity and fuse this to an immunoglobulin Fc domain, creating a therapeutic (ALPN-202, davoceticept) capable of providing CD28 costimulation in a PD-L1-dependent fashion while also antagonizing PD-1 - PD-L1 and CTLA-4–CD80/CD86 interactions. We demonstrate that by combining CD28 costimulation and dual checkpoint inhibition, ALPN-202 enhances T cell activation and anti-tumor efficacy in cell-based assays and mouse tumor models more potently than checkpoint blockade alone and thus has the potential to generate potent, clinically meaningful anti-tumor immunity in humans.

## Abstract (web summary)

CD28 costimulatory signalling can be suppressed by immune checkpoints, such as CTLA-4 and PD-1. Here the authors describe the design of the fusion therapeutic davoceticept (ALPN-202), based on a variant CD80 extracellular domain engineered to bind PD-L1 as well as CD28 and CTLA-4, providing direct T cell costimulation and dual checkpoint inhibition to enable anti-tumor immune responses.

## Introduction

Immune checkpoint inhibitors (CPI) targeting the CTLA-4—CD80/CD86 and PD-1–PD-L1 pathways have demonstrated significant clinical activity in many cancers, either as monotherapy or in combination with current standard of care regimens^1^. Despite the impressive clinical outcomes achieved for some cancers, many patients fail to respond to CPI entirely, or the observed response lacks durability due to the development of acquired resistance^2^. Primary and acquired resistance to immunotherapy is the subject of intensive research, with several proposed tumor-intrinsic and extrinsic mechanisms implicated, including increased expression of metabolic mediators, impaired antigen presentation and T cell activation, recruitment of immunosuppressive cells, modulation of immune checkpoints, and impaired IFNγ signaling, among others^3^. To overcome these obstacles, rationally designed combinations of drugs targeting multiple inhibitory and costimulatory pathways will likely be required. In support of this concept, preclinical data demonstrating the distinct and synergistic mechanisms of CTLA-4 and PD-1 blockade^4,5^ have translated into improved clinical activity and patient outcomes in randomized trials in melanoma and renal cell carcinoma^6,7^. More recently, several groups have demonstrated that CTLA-4 and PD-1 inhibition works either directly or indirectly through suppression of CD28 costimulatory signaling^8–10^ and lack of sufficient T cell costimulation in the tumor microenvironment may be involved in primary or acquired resistance to CPI^11–15^. This raises the possibility that an approach combining CPI with CD28 costimulation could be more potent than CPI alone.

Intriguingly, CD80 not only has a well-documented role modulating T cell responses via the CTLA-4 and CD28 co-receptors, but also recently has been demonstrated to bind PD-L1 *in cis* on the surface of antigen-presenting cells (APC)^16,17^. While the significance of the CD80–PD-L1 interaction to the frequency and durability of clinical responses to CPI is still under active investigation, the importance of this family of proteins to the regulation of immune responses makes this interaction of high interest. Recent data suggest that CD80, when bound to PD-L1 *in cis*, appears to retain the ability to costimulate CD28 while blocking PD-L1 engagement with PD-1^18,19^, suggesting that the relative expression levels of each of these receptors likely contributes to the overall costimulatory/coinhibitory activity of an APC^20^.

Building on these observations, we report herein the use of a yeast surface display directed evolution platform^21^ to selectively engineer a CD80 IgV domain for increased affinity for PD-L1 and CD28 relative to wild type (WT) CD80, while maintaining the ability to bind CTLA-4. The selected CD80 IgV domain is fused to an inert Fc domain to become a tri-specific therapeutic agent, ALPN-202 (davoceticept), capable of antagonizing CTLA-4 and PD-1 and delivering a PD-L1-dependent T cell costimulatory signal, and potentially improving upon CPI standard of care therapies in multiple oncology indications.

## Results

### Engineering of CD80 via directed evolution

**Fig. 1:**
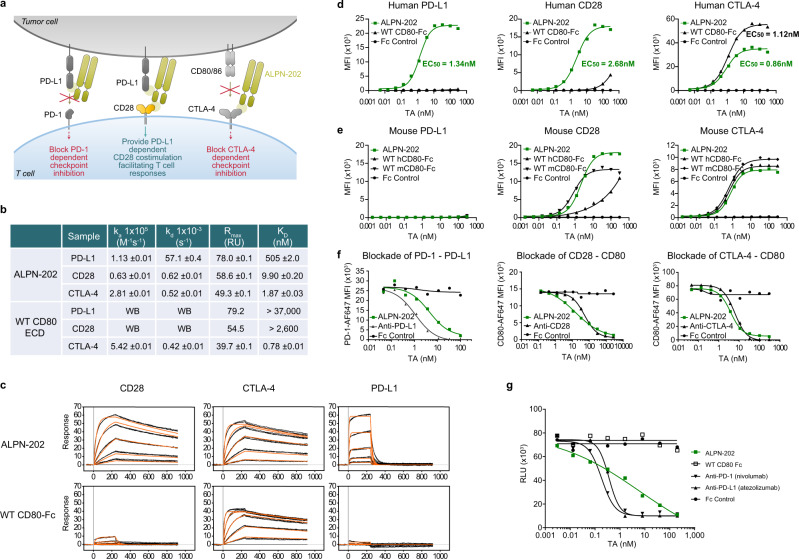
ALPN-202, a variant CD80 IgV domain Fc fusion, binds human PD-L1, CD28, and CTLA-4 with high affinity. **a** Schematic illustrating the three mechanisms of action of ALPN-202: blockade of PD-1–PD-L1 interaction, PD-L1-dependent CD28 costimulation, and blockade of CTLA-4–CD80/CD86 interactions. **b**, **c** Affinity measurements of ALPN-202 and WT CD80-Fc to monomeric wild type PD-L1, CD28, and CTLA-4 were determined by surface plasmon resonance (SPR). Sensorgrams are shown in black lines and results from non-linear least squares regression analysis of the data in orange lines. Sensorgrams were global fit to a 1:1 binding model for triplicate injections of human CD28, CTLA-4, and PD-L1 against captured ALPN-202 and WT CD80-Fc surfaces. For the weak CD28–WT CD80-Fc and PD- L1–WT CD80-Fc interactions, the theoretical Rmax was used as a fixed parameter in the global fit to estimate the KD. WB, weak binding; WT, wild type; ECD, extracellular domain. **d**, **e** ALPN-202 binding to CHO cells stably expressing human or mouse PD-L1, CD28, or CTLA-4. ALPN-202 displayed higher affinity for human PD-L1 and CD28, and comparable affinity for CTLA-4, relative to WT CD80-Fc. ALPN-202 did not bind to mouse PD-L1 but did bind mouse CD28 and CTLA-4 with comparable affinity as WT mouse CD80-Fc. TA test article; MFI median fluorescent intensity. **f** ALPN-202 blocked binding of PD-1-AF647 to cell surface PD-L1 and CD80-AF647 to cell surface CD28 and CTLA-4 to CD80 measured by flow cytometry. **g** ALPN-202 blocked human PD-L1-mediated recruitment of SHP-2 to PD-1 in a cell-based assay. RLU, relative luminescence units. Experiments in (**d**–**g**) were conducted two times and data shown are representative. Source data are provided as a Source Data file.

The WT CD80 extracellular domain (ECD) is comprised of a membrane-proximal IgC domain and a distal IgV domain. To engineer variant CD80 Ig domains (vIgDs) with high affinity for PD-L1, we generated CD80 ECD and IgV-only libraries via error-prone PCR and screened the novel variants using yeast surface display as described^21^. Screening consisted of sorting and selection by binding the CD80 vIgDs to varying concentrations of Fc-dimers of CTLA-4, CD28, and PD-L1. Multiple rounds of library generation and sorting, with further mutagenesis between rounds, were conducted to generate a panel of CD80 vIgDs with improved affinity for PD-L1 and CD28. The vIgDs were fused to Fc-coding regions containing previously described mutations to reduce Fc receptor interactions^22,23^, transfected into mammalian HEK293 cells, expressed, and purified as CD80 vIgD Fc-fusion proteins. The CD80 vIgDs were screened for binding to PD-L1, CD28, and CTLA-4, and tested for in vitro and in vivo function (Supplementary Fig. 1). An IgV-only CD80 vIgD Fc-fusion protein with higher affinity for both PD-L1 and CD28 relative to WT CD80 ECD was identified and designated ALPN-202 (Fig. 1a).

### ALPN- 202 binds PD-L1 with higher affinity than WT CD80 and blocks PD-1 interaction

The binding affinity of ALPN-202 to its three counter-structures, PD-L1, CD28, and CTLA-4, was determined by surface plasmon resonance (SPR) (Fig. 1b) and compared to WT CD80 ECD-Fc. The affinity of ALPN-202 for PD-L1, CD28, and CTLA-4 was 505 nM, 9.9 nM, and 1.87 nM, respectively, under the experimental conditions used. ALPN-202 binding to monomeric PD-L1 demonstrated a rapid on-rate, as well as a relatively rapid off-rate (Fig. 1c). The WT CD80 ECD-Fc binding K_D_ for CTLA-4 was 0.78 nM, slightly higher than ALPN-202, while the interactions with PD-L1 and CD28 were relatively weak with affinities estimated at >37,000 nM and >2600 nM, respectively.

ALPN-202 binding to cell-surface human (Fig. 1d) and mouse (Fig. 1e) PD-L1, CD28, and CTLA-4, on transduced Chinese hamster ovary (CHO) cells was measured by flow cytometry and compared to that of WT human or mouse CD80 ECD-Fc. Despite the off-rate observed by SPR, ALPN-202 binding to PD-L1^+^ cells was dramatically increased relative to WT CD80 ECD-Fc (EC50 of 1.34 nM and out of range (OOR) respectively) suggesting that avidity plays a factor in increasing the duration of interaction with PD-L1. In agreement with the SPR data, the binding affinity of ALPN-202 for CD28 was also increased relative to that of WT CD80 ECD-Fc (EC50 values of 2.68 nM and >100 nM, respectively). Lastly, ALPN-202 bound CTLA-4 with comparable affinity as WT CD80 ECD-Fc (EC50 values of 0.86 nM and 1.12 nM respectively), but with a lower maximum mean fluorescence intensity (MFI). We speculate that the differences in observed binding to CD28 and CTLA-4 by ALPN-202 may be due to differences in the dimerization behavior caused by removing the IgC domain and inclusion of a mutation in the CD80 vIgD near the CD28/CTLA-4 binding site. Overall, this binding profile to the human counter-structures is consistent with the positive selection scheme used during the directed mutagenesis and variant selection. When we assessed binding to mouse counter-structures, we observed that ALPN-202 bound CD28 and CTLA-4 with EC50 values comparable to WT mouse CD80 ECD-Fc, but there was no measurable binding to mouse PD-L1 (Fig. 1e).

The ability of ALPN-202 to block PD-1–PD-L1, CD80–CTLA-4, and CD80–CD28 interactions was tested by flow cytometry and compared to blocking antibodies to PD-L1 (atezolizumab), CD28, and CTLA-4 (ipilimumab) (Fig. 1f). ALPN-202-mediated blockade of WT PD-1-Fc binding to PD-L1 was slightly weaker than atezolizumab. ALPN-202-mediated blockade of WT CD80 interactions with CD28 and CTLA-4 was comparable to the CD28 and CTLA-4 antibodies, respectively.

To further confirm that ALPN-202 functionally blocks the PD-1–PD-L1 interaction, we ran a cell-based assay measuring recruitment of SHP-2 to the PD-1 intracellular domain following ligation with PD-L1 (Fig. 1g). ALPN-202 bound PD-L1 and inhibited SHP-2 recruitment in a dose-dependent manner, though with a higher EC50 than either anti-PD-1 (nivolumab) or anti-PD-L1 (atezolizumab) (6.24 nM, 0.18 nM, and 0.36 nM, respectively) suggesting that ALPN-202 may not block PD-1–PD-L1 interaction as potently as either antibody, at least in this assay.

### Characterization of PD-L1-dependent CD28 costimulation

**Fig. 2:**
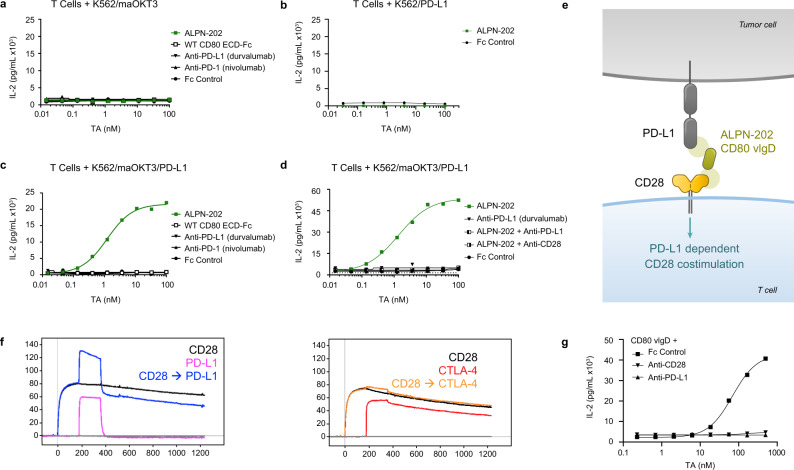
CD28 costimulation by ALPN-202 requires TCR activation and co-binding to PD-L1. Primary human T cells, were co-cultured for 24 h with test articles (TA) and K562 cell artificial antigen-presenting cells (aAPC) expressing membrane anchored anti-CD3 clone OKT3 (maOKT3), PD-L1, or both. **a** In the absence of PD-L1 to anchor ALPN-202 on the aAPC, no increase in IL-2 was detected above background. **b** In the absence of TCR stimulation (via maOKT3), no increase in IL-2 was observed. **c** When both maOKT3 and PD-L1 were present on the aAPC, ALPN-202 (green line) induced a strong dose-dependent costimulatory signal above that of WT CD80 ECD-Fc or PD-(L)1 blockade alone. WT wild type; ECD extracellular domain. **d** The costimulatory activity of ALPN-202 was inhibited when combined with blocking antibodies to either PD-L1 or CD28, confirming the requirement for dual binding to induce costimulation. **e** Schematic illustrating how a monomeric (no Fc) CD80 variant Ig domain (vIgD) binds PD-L1 and engages CD28 in trans. **f** Overlaid SPR sensorgrams for two paired serial injections of monomeric CD28 (black line) followed by PD-L1 (blue line) or CTLA-4 (orange line) against ALPN-202 surfaces. Data were collected using two serial 180 s, 1500 nM injections. Single analyte injections of PD-L1 (pink line) or CTLA-4 (red line) were made by pairing with blank buffer injections. **g** Primary T cells were co-cultured with K562/maOKT3/PD-L1 cells and a titration of the monomeric ALPN-202 CD80 vIgD with saturating anti-PD-L1 antibody, anti-CD28 antibody, or Fc control for 24 h. For (**a**–**d**), and (**f**), each data point is mean concentration of IL-2 for each sample run in duplicate wells. Data shown are representative of three separate donors run independently. Source data are provided as a Source Data file.

Given that ALPN-202 binds PD-L1 with higher affinity than WT CD80 ECD-Fc, we developed a primary T cell activation assay that employs an artificial APC (aAPC) comprised of K562 cells expressing either membrane-anchored anti-CD3 single-chain Fv (maOKT3)^24^, full-length human PD-L1, or both. Co-culturing primary T cells with different aAPCs allowed us to characterize the capacity of ALPN-202 to induce PD-L1-dependent CD28 costimulation. When the aAPC expressed only maOKT3, ALPN-202 did not increase IL-2 production by T cells above TCR stimulation alone (Fig. 2a). To demonstrate that ALPN-202 activity was costimulatory as opposed to overtly stimulatory (i.e., a CD28 superagonist), we co-cultured T cells with aAPCs expressing PD-L1 but lacking maOKT3. In this experiment, ALPN-202 failed to induce IL-2, demonstrating that a T cell receptor (TCR) signal is required to enable the PD-L1-dependent CD28 costimulation (Fig. 2b). However, when the aAPC expressed both maOKT3 and PD-L1, ALPN-202 strongly enhanced IL-2 production (Fig. 2c). The level of IL-2 production was significantly increased relative to PD-1–PD-L1 blockade alone, suggesting more than simple blockade of an inhibitory signal. To demonstrate that the observed activity requires simultaneous binding of CD28 on the T cells and PD-L1 on the aAPC, we combined ALPN-202 with either blocking antibodies to CD28 or PD-L1 (Fig. 2d). In both cases, ALPN-202-mediated IL-2 induction was potently inhibited.

We additionally conducted cytokine release assays developed to predict the CD28 superagonist activity of therapeutics in preclinical development^25,26^. In these assays, incubation of ALPN-202 (either plate bound or in solution) with high-density PBMC cultures from multiple donors failed to induce any cytokine release above negative control molecules (Supplementary Fig. 2).

To more fully characterize its costimulatory activity, we tested ALPN-202 at submaximal TCR stimulation by co-culturing T cells with K562/PD-L1 cells and a titration of anti-CD3 and compared activity to anti-PD-1, anti-CTLA-4, or agonist anti-CD28. In addition to secreted IL-2, we examined intracellular IL-2 production and upregulation of CD25 expression, a commonly used activation marker responsive to CD28 costimulation (Supplementary Fig. 3). ALPN-202 increased secreted and intracellular IL-2 as well as upregulated CD25 in a dose- and anti-CD3-dependent manner comparable to the control CD28 agonist antibody. In all cases, ALPN-202 induced a stronger response than anti-PD-L1, anti-CTLA-4, or dual CPI, consistent with the costimulatory activity of ALPN-202.

### Monomeric CD80 vIgDs can simultaneously bind CD28 and PD-L1

Recent publications have suggested that when CD80 is bound to PD-L1 in *cis*, it maintains the ability to bind CD28 in *trans* and induce costimulatory activit*y*^18,19^. To confirm these findings and to better understand the ALPN-202 mechanism of action, we sought to determine if a single CD80 IgV domain could simultaneously bind PD-L1 and CD28 and induce a PD-L1-dependent CD28 costimulatory signal (Fig. 2e). Using SPR, ALPN-202 was captured to a sensor chip and then sequentially co-injected with CD28 followed by PD-L1 or CTLA-4 (Fig. 2f). PD-L1, but not CTLA-4, bound ALPN-202 in the presence of saturating amounts of CD28, indicating separate binding surfaces.

To test if a single CD80 vIgD (no Fc) was able to induce a PD-L1-dependent CD28 costimulatory signal, primary human T cells were co-cultured with an aAPC expressing maOKT3 and PD-L1 for 24 h, and a titration of monomeric CD80 vIgD in the presence of saturating anti-CD28 or anti-PD-L1 (Fig. 2g). The monomeric CD80 vIgD induced IL-2 in a dose-dependent manner, indicating CD28 costimulatory activity. As observed with intact ALPN-202, combining the monomeric CD80 vIgD with anti-PD-L1 or anti-CD28 completely abrograted the induction of IL-2 above TCR stimulation alone.

### Crystal structure of ALPN-202 vIgD:PD-L1 complex

**Fig. 3:**
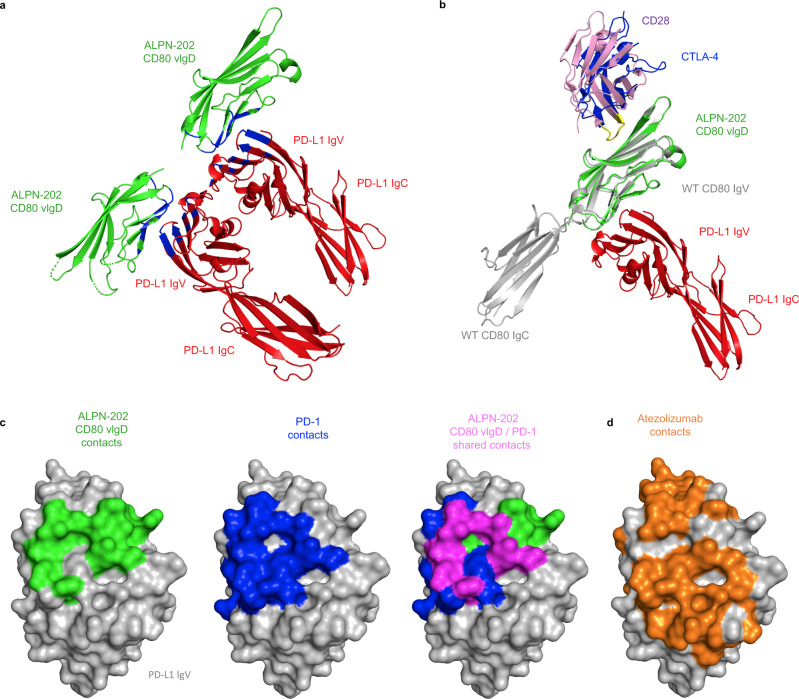
Crystal Structure of ALPN-202 CD80 vIgD complexed with the wild type PD-L1 ECD. **a** Representative ribbon structure from the ALPN-202 CD80 variant Ig domain (vIgD) (green) and wild type (WT) PD-L1 extracellular domain (ECD) (red) crystal structure with the interface contact residues indicated in blue (PDB: 7TPS) **b** Model alignments of one subunit (Chain A and Chain B) of the ALPN-202 CD80 vIgD (green)/PD-L1 ECD (red) asymmetric unit aligned with wild type CD80 ECD (gray)/CTLA-4 ECD (blue) (PDB: 1I8L) and CD28 (pink) (PDB: 1YJD). The CD28 and CTLA-4 MYPPPY motifs are indicated in yellow. **c** Comparison of ALPN-202 CD80 vIgD binding contacts in green, PD-1 binding contacts in blue (PDB: 4ZQK) and ALPN-202 and PD-1 common contacts indicated in magenta mapped onto the surface of PD-L1 IgV domain (gray). **d** Atezolizumab (orange) contacts mapped to the same surface as PD-1 and ALPN-202 CD80 vIgD contact residues on the surface of PD-L1 IgV (gray).

The CD80 vIgD from ALPN-202 was co-crystallized with WT PD-L1 ECD and used to determine the structure of the CD80 vIgD:PD-L1 complex (PDB: 7TPS). The structure shows that the CD80 vIgD interacts with the IgV domain of the PD-L1 ECD (Fig. 3a, Supplementary Table 1). Alignment of the CD80 vIgD:PD-L1 structure with the existing structure of the CD80:CTLA-4 binding complex (PDB: 1I8L), and aligning CD28 (PDB: 1YJD) to CTLA-4 using the MYPPPY motif^27,28^ as an anchor, demonstrates that the CD80 vIgD binds PD-L1 on the face opposite the binding site of CTLA-4 and CD28 (Fig. 3b). Thus, the CD80 vIgD domain of ALPN-202 appears capable of simultaneously binding both PD-L1 and either CD28 or CTLA-4, consistent with the binding and functional activity shown above.

The contact surface between PD-L1 and the CD80 vIgD overlays the contact surface of PD-L1 and PD-1, demonstrating how ALPN-202 blocks PD-1–PD-L1 binding (Fig. 3c). In all, PD-1 makes 17 contacts with PD-L1 (PDB: 4ZQK) and the CD80 vIgD from ALPN-202 makes 13 contacts with PD-L1, with 7 of these being shared residues (highlighted in purple, Fig. 3c). Atezolizumab contact residues on PD-L1^29^ (Fig. 3d) indicate that the ALPN-202 CD80 vIgD contacts PD-L1 at an overlapping epitope.

The ALPN-202 CD80 vIgD contains seven amino acid substitutions located throughout the CD80 IgV domain (Supplementary Fig. 4a), including sites at or near the known IgV-IgC interface, the IgV–IgV homodimer interface^30^, and the CTLA-4/CD28 binding interface^31–33^ (Supplementary Fig. 4b). In an effort to understand the functional relevance of each mutation, we produced a set of CD80 vIgDs starting with wild-type CD80 where each of the mutations was added alone or in combination. A subset of these proteins was then assessed for changes in binding affinity (Supplementary Fig. 4c). Some mutations specifically affect binding to PD-L1 (i.e. E35D and M47L) while others appear to contribute in combination (i.e. V68M). Some of the other mutations did not appear to directly influence binding to PD-L1 and may contribute more to protein stability or CD28 and CTLA-4 binding. Additional work is needed to more fully understand how each mutation contributes individually and in combination to the activity and stability of ALPN-202.

### PD-L1 on M2c macrophages enables ALPN-202-mediated CD28 costimulation

**Fig. 4:**
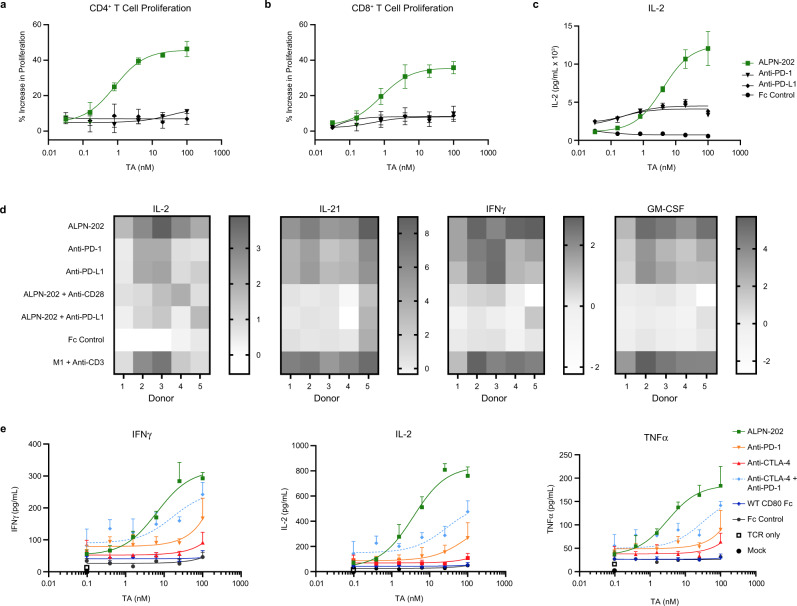
ALPN-202 increases T cell activation more potently than CPI alone in the presence of PD-L1^+^ suppressive M2c macrophages. Primary human T cells were co-cultured with in vitro-derived M2c macrophages and a titration of ALPN-202 or anti-PD-1, PD-L1, or CTLA-4 blocking antibodies. ALPN-202 induced (**a**) CD4^+^ T cell proliferation, (**b**) CD8^+^ T cell proliferation, and (**c**) IL-2 secretion in a dose-dependent manner more potently than CPI alone. Proliferation data shown are mean percent increase relative to Fc control and is from a representative donor (*n* = 5 donors). Error bars are SD from triplicate wells. TA test article. **d** ALPN-202 activity increased expression of IL-2, IL-21, IFNγ, and GM-CSF in cell culture supernatants and is dependent on binding to PD-L1 and CD28. Data are shown as fold-increase (dark shade) or decrease (white) with respect to untreated control (M2c + anti-CD3). Data shown are from five donors and were collected in two independent experiments. M1 proinflammatory M1 macrophages. **e** To compare ALPN-202 activity to PD-1, CTLA-4, or dual checkpoint inhibition (CPI), E6 TCR^+^ T cells were co-cultured with M2c macrophage and SCC152/hPD-L1^+^ tumor cells for 24 h. Mean ± SD for IFNγ, IL-2, and TNFα was quantitated from triplicate wells. Data are representative of four donors tested in two independent experiments. E6 TCR—T cell receptor specific for E6 peptide. Source data are provided as a Source Data file.

Many tumors are highly infiltrated by CD163^+^ M2c pro-tumorigenic macrophages which actively suppress anti-tumor immune responses by: upregulation of PD-L1, downregulation of CD80/86, and release immunosuppressive cytokines and metabolism-altering factors like IDO and NO^13,34,35^. To determine if endogenously expressed PD-L1 on M2c macrophages is sufficient to enable ALPN-202-mediated CD28 costimulation, we developed an in vitro co-culture assay combining primary T cells, sub-maximal anti-CD3 antibody and in vitro-differentiated PD-L1^+^ M2c macrophages expressing little to no CD80 or CD86 (Supplementary Fig. 5). In this assay, ALPN-202 potently increased CD4^+^ and CD8^+^ T cell proliferation and IL-2 production (Fig. 4a–c) more potently than blockade of the PD-1–PD-L1 pathway alone. Additionally, when combined with anti-CD28 or anti-PD-L1 blocking antibodies, the costimulatory activity of ALPN-202 was significantly abrogated, suggesting that, consistent with our proposed mechanism of action, the enhanced response required simultaneous binding of both PD-L1 and CD28 (Fig. 4d).

To compare ALPN-202 to dual PD-1 and CTLA-4 checkpoint blockade in an in vitro setting, we co-cultured primary human E6 TCR transgenic T cells (human papilloma virus (HPV) E6 peptide-specific) with autologous M2c macrophages and PD-L1-expressing SCC152 human tumor cells at a 1:1:1 ratio. (Fig. 4e). In all four donors tested, ALPN-202 induced elevated IFNγ, IL-2, and TNFα in a dose-dependent manner more effectively than single or double checkpoint blockade alone, demonstrating the CD28 costimulatory activity of ALPN-202.

### ALPN-202 monotherapy demonstrates potent anti-tumor activity in vivo

**Fig. 5:**
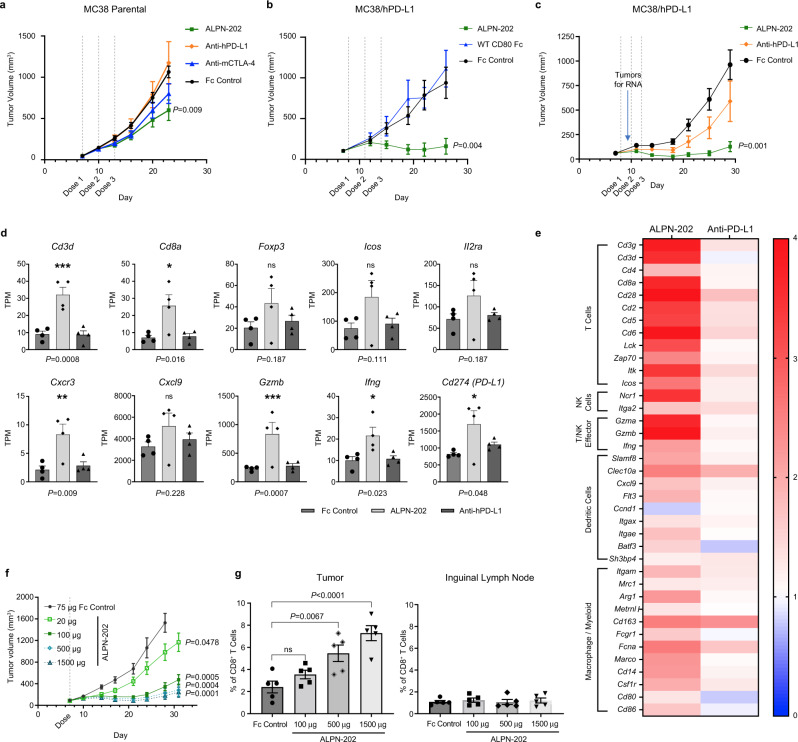
ALPN-202 demonstrates potent and dose-dependent anti-tumor activity in vivo in a MC38/human PD-L1 tumor model. **a** ALPN-202 induced a small but significant decrease in tumor growth relative to Fc control in a parental MC38 tumor model. (*n* = 10 mice/group). **b**, **c** In MC38/hPD-L1 tumor studies, ALPN-202-mediated anti-tumor activity (green) was superior to wild type WT CD80 ECD-Fc (blue), anti-PD-L1 (durvalumab, orange), or isotype control (black) (*n* = 10 mice/group). WT wild type; ECD extracellular domain. **d** A subset of tumors from study shown in (**c**) were harvested 72 h after the first dose and RNAseq performed. Transcripts per kilobase million reads (TPM) of select genes for individual tumors are shown. Individual points represent TPM for individual tumor samples and bars indicate mean values ± SEM. **e** Signature transcripts from the indicated immune cell populations were identified using the ImmGen Population Comparison application [https://www.immgen.org/ImmGenpubs.html]. The heatmap compares the fold increase (red) or decrease (blue) in mean transcript levels of either ALPN-202-treated or anti-PD-L1 treated tumors relative to Fc-control-treated tumors (*n* = 4 tumor samples per group). **f** ALPN-202-treated MC38/hPD-L1 tumors (*n* = 12 mice/group) showed a dose-dependent increase in anti-tumor activity. **g** 72 h after treatment there was a dose-dependent increase in intratumoral p15E^+^ tumor antigen-specific CD8^+^ T cells. No change was observed in inguinal lymph nodes. *N* = 5 samples from each group (individual symbols) and bar height indicates mean percentage of p15E^+^ CD8^+^ T cells. Error bars are SEM. Gating strategy is summarized in Supplementary Fig. 7. Statistical significance was determined by one-way ANOVA with Dunnett’s multiple comparisons test (**d**, **g**) or by repeated-measures two-way ANOVA for treatment effects (**a**, **b**, **c**, **f**). *P* values shown are ALPN-202 vs Fc control. For all studies except (**f**, **g**), antibodies and ALPN-202 were treated at 100 µg/dose while Fc control was 75 µg/dose. For all graphs, the means ± SEM are shown. Source data are provided as a Source Data file.

The anti-tumor activity of ALPN-202 was initially tested in a syngeneic MC38 mouse colorectal adenocarcinoma solid tumor model (Fig. 5a). A small but significant decrease in tumor growth, comparable to CTLA-4 blockade alone, was observed. We hypothesized that ALPN-202 was unable to induce CD28 costimulation in this model because of its inability to bind mouse PD-L1 and thus the anti-tumor activity was only due to ALPN-202’s ability to bind and block CTLA-4. To address this, we stably transduced MC38 cells with human PD-L1 (MC38/hPD-L1) and used these cells to study the mechanistic activity of ALPN-202 in vivo. Using this model, the anti-tumor activity of ALPN-202 was significantly improved relative to WT CD80 Fc (Fig. 5b) or anti-human PD-L1 treatment (Fig. 5c).

### Characterization of ALPN-202 activity in the tumor microenvironment

To more comprehensively understand ALPN-202-induced changes within the tumor, we profiled changes in gene expression by RNAseq on MC38/hPD-L1 tumors 72 h after a single dose of ALPN-202, anti-PD-L1, or Fc control (Fig. 5c). Anti-PD-L1 treated tumors were not statistically different from controls; however, ALPN-202-treated tumors showed increased expression of genes associated with an effective T cell-driven anti-tumor response (Fig. 5d). In addition to significantly increased T cell lineage transcripts (e.g., *Cd3d* and *Cd8a*) and effector genes (e.g., *Gzmb* and *Ifng*), we also observed a trend toward increased T cell activation markers (e.g., *Icos*, *Il2ra*). One mouse in the ALPN-202 treated group did not show elevated T cell activation or other markers of immune response and we speculate that this animal may not have received a full dose of ALPN-202. There was also an increase in a broad array of dendritic cell- and macrophage-related transcripts (Fig. 5e) including chemokines known to recruit T and NK cells (e.g., *Cxcl9*). We hypothesize that the increased frequency of myeloid lineage transcripts was indirect, and reflected a response to T cell-produced IFNγ and TNFα, though additional work is needed to clearly understand these results. Enricher Pathway analysis^36,37^ of the top 124 gene expression changes induced in ALPN-202-treated tumors relative to Fc-control treated tumors showed strong correlations with multiple T cell and other immune cell activation pathways, including T cell receptor stimulation and costimulation gene signatures (Supplementary Data 1), supporting the proposed mechanism of action for ALPN-202.

The dose–response relationship of ALPN-202 in the MC38/hPD-L1 tumor model was investigated (Fig. 5f). ALPN-202, administered as a single IP injection, resulted in a dose-dependent increase in serum exposure (Supplementary Fig. 6) and significant anti-tumor activity even at the lowest dose tested (20 μg).

To understand how ALPN-202 mediates anti-tumor responses in this model, tumors and inguinal lymph nodes were excised from a subset of mice 72 h after treatment with ALPN-202 or Fc control, and tumor-infiltrating immune cells were characterized by flow cytometry. We used the mouse MHC class I p15E tetramer, which recognizes a tumor antigen expressed by MC38 cells, to demonstrate a dose-dependent increase in the percentage of infiltrating tumor antigen-specific CD8^+^ T cells (Fig. 5g). This effect appears limited to the tumor microenvironment, as no change was observed in the percentage of p15E tetramer^+^ T cells in the inguinal lymph nodes tested.

### PD-L1 and CD28 engagement for maximal anti-tumor activity in vivo

**Fig. 6:**
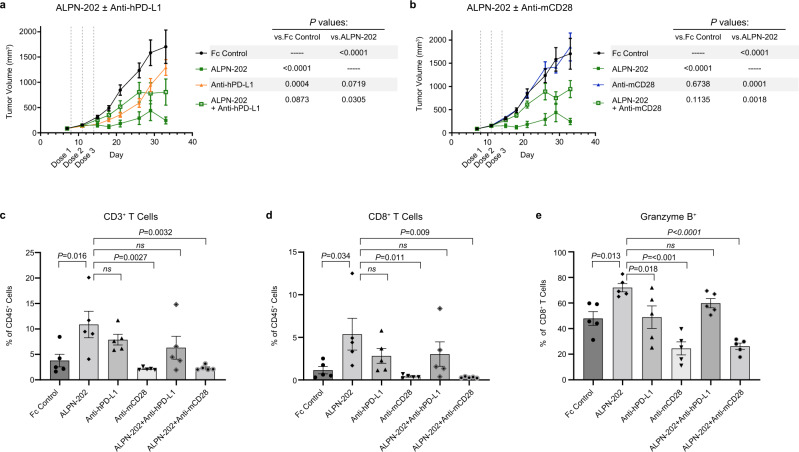
ALPN-202 increases intratumoral inflammatory cell infiltrate in part by hPD-L1-dependent mCD28 costimulation. **a**, **b** The anti-tumor activity of ALPN-202 was significantly blocked by combining with (**a**) anti-hPD-L1 or (**b**) anti-mCD28 blocking antibodies (*n* = 12 mice/group). Mean tumor volumes ± SEM are shown. Tumors were collected from mice 48 h after the second dose, dissociated, and analyzed by flow cytometry. ALPN-202 treatment significantly increased the percentage of infiltrating total CD3^+^ T cells (**c**), CD8^+^ T cells (**d**), and granzyme B^+^ CD8^+^ T cells (**e**) relative to Fc Control treated animals. In each case, this influx in T cells was reduced when ALPN-202 was combined with anti-hPD-L1 or anti-mCD28 antibodies. Statistical significance was determined by repeated-measures two-way ANOVA for treatment effects (**a**, **b**) or one-way ANOVA with Dunnett’s multiple comparisons test (**c**–**e**). Bar height indicates mean value ± SEM and symbols represent individual tumor samples (*n* = 5 tumors per treatment group) (**c**–**e**). Source data are provided as a Source Data file.

To demonstrate that ALPN-202 anti-tumor activity is at least partially mediated by PD-L1-dependent CD28 costimulation and is dependent on simultaneous engagement of both PD-L1 and CD28, we treated MC38/hPD-L1 tumor-bearing mice with ALPN-202 alone or in combination with blocking antibodies to mouse CD28 (clone E18) or human PD-L1 (durvalumab). ALPN-202 treatment induced a significant reduction in tumor growth (Fig. 6a) that was superior to that of PD-L1 blockade. When ALPN-202 was combined with PD-L1 blockade however, anti-tumor activity was reduced, suggesting that ALPN-202 requires PD-L1 binding for anti-tumor activity. No significant change in tumor growth was observed when mice were treated with an anti-CD28 blocking antibody (Fig. 6b). When ALPN-202 was combined with anti-CD28, anti-tumor activity was significantly reduced compared to ALPN-202 administration alone (Fig. 6b). Collectively, these results demonstrate that PD-L1-dependent CD28 costimulation is at least partially responsible for the anti-tumor activity of ALPN-202 in this model.

Forty-eight hours after the second dose, tumors from a cohort of mice were excised, dissociated, and analyzed by flow cytometry. ALPN-202 significantly increased total T cell infiltration (Fig. 6c), CD8^+^ T cell infiltration (Fig. 6d), and the percentage of granzyme B^+^ CD8^+^ T cells (Fig. 6e) versus Fc control. The percentage of cellular infiltrates was substantially reduced when ALPN-202 was combined with either anti-PD-L1 or anti-CD28 blocking antibodies, again suggesting that ALPN-202-mediated anti-tumor activity is dependent on PD-L1-dependent CD28 costimulation leading to increased T cell infiltration and activation.

### ALPN-202 cooperates with anti-mouse PD-1 for improved anti-tumor activity

**Fig. 7:**
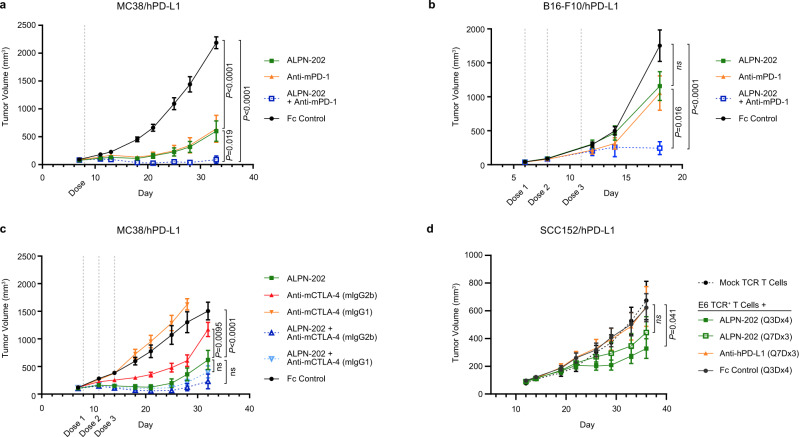
ALPN-202 combines with anti-PD1 and anti-CTLA-4 antibodies and is active in a humanized tumor model. **a**, **b** Tumor growth curves for (**a**) hPD-L1-expressing MC38 tumors (*n* = 10 mice/group) or (**b**) B16-F10 tumors (*n* = 9 mice/group) treated with ALPN-202 (green line), anti-mPD-1 (orange line), a combination of ALPN-202 and anti-mPD-1 (blue), or an isotype control (black). **c** MC38/hPD-L1 tumors (*n* = 9 mice/group) treated with ALPN-202 (green), anti-mCTLA-4 formatted with either mIgG2b Fc (red) or inert Fc (orange), or combinations of ALPN-202 with each mCTLA-4 antibody (dark blue, light blue respectively). **d** Tumor growth curves (*n* = 9 mice/group) from NSG mice implanted with human E6 TCR^+^ transgenic T cells as well as human SCC152/hPD-L1 tumor cells treated with ALPN-202 either every three days for four doses (dark green; Q3Dx4) or once every seven days for three doses (light green; Q7Dx3), anti-hPD-L1 (durvalumab, orange) or Fc control (black). NSG NOD-scid IL2Rγ^null^; E6 TCR, T cell receptor specific for E6 peptide. All experiments were repeated at least two times and data shown are representative. Statistical significance was determined by repeated-measures two-way ANOVA for treatment effects. For all graphs, means ± SEM are shown. Source data are provided as a Source Data file.

Because ALPN-202 does not block the mouse PD-1–PD-L1 interaction, we sought to understand how hPD-L1-mediated CD28 costimulation would compare to or work in combination with mPD-1 blockade in two separate tumor models: MC38/hPD-L1 (Fig. 7a) and the more immunotherapy-resistant B16-F10/hPD-L1 (Fig. 7b). In each model, we tested anti-mPD-1 and ALPN-202 alone or in combination. In both models, each agent alone provided roughly equivalent anti-tumor activity; however, there was a highly significant increase in anti-tumor activity when both agents were combined.

### ALPN-202 with Treg-depleting CTLA-4 antibodies improves anti-tumor responses

In mouse models, the anti-tumor efficacy of CTLA-4 antibodies has been linked to their ability to deplete CTLA-4^+^ Treg cells^38^. However, because ALPN-202 was engineered to lack FcR binding, we did not expect depletion of CTLA-4^+^ cells in vivo. We therefore tested whether ALPN-202-mediated anti-tumor activity would be improved when combined with depleting or non-depleting variants of anti-CTLA-4 in the MC38/hPD-L1 model (Fig. 7c). While the inert Fc mCTLA-4 antibody had no anti-tumor activity, the anti-CTLA-4 mIgG2b Fc (which can mediate ADCC) showed a moderate decrease in tumor volume. ALPN-202 had greater anti-tumor efficacy than either antibody alone and this activity was slightly potentiated when combined with anti-CTLA-4 mIgG2b.

### ALPN-202 improves the anti-tumor response of human TCR Tg T cells in vivo

To ensure that ALPN-202 anti-tumor activity translates to a humanized model, we implanted SCC152/PD-L1 tumor cells in immunocompromised NSG mice infused with E6 TCR^+^ T cells. Following the Tg TCR T cell transfer, ALPN-202 was dosed using two different regimens, every three days for four doses (Q3Dx4), or every seven days for three doses (Q7Dx3) and compared to anti-PD-L1 (durvalumab) dosed Q7Dx3 (Fig. 7d). ALPN-202 dosed Q3Dx4 showed a significant decrease in tumor volume, and a trend towards lower tumor volume was observed when ALPN-202 was dosed Q7D. No anti-tumor response was observed in anti-PD-L1-treated mice.

## Discussion

PD-1 and CTLA-4 antagonists have revolutionized the treatment of multiple tumor types by increasing the quality of T cell priming by DCs, improving expansion of stem-like T cells, and ‘reviving’ exhausted tumor antigen-specific T cell responses^39–41^. However, despite our improved understanding of the mechanistic basis of these therapies, the majority of patients still do not achieve lasting remissions and it has been suggested that one contributing factor is a lack of robust T cell costimulation^9,10^. We therefore sought to develop a therapeutic entity that could combine checkpoint inhibition and T cell costimulation to improve the frequency and durability of anti-tumor responses.

Inspired by recent observations that CD80 binds PD-L1 *in cis* and prevents interaction with PD-1^16–18^, we generated a variant CD80 IgV-Fc protein, ALPN-202, with improved affinity for PD-L1. We demonstrate that not only does ALPN-202 bind CTLA-4 and PD-L1 with high affinity, it also provides a PD-L1-dependent CD28 costimulatory signal to T cells in trans leading to increased T cell proliferation and cytokine production when compared to CPI alone. It should be noted that while the affinity of ALPN-202 for PD-L1 is significantly higher than WT CD80, the *K*_D_ is lower than that of therapeutic PD-1 and PD-L1 antibodies^42^ and more experiments are required to evaluate the relative potency of ALPN-202 to block the PD-1–PD-L1 interaction in humanized in vivo tumor models.

To better understand the PD-L1–CD80 interaction, we co-crystallized the CD80 IgV domain from ALPN-202 complexed with a PD-L1 ECD and solved a CD80:PD-L1 structure. PD-L1 binds CD80 on a surface that does not overlap with the CD28–CTLA-4 binding site (Fig. 3b). These data are consistent with recent publications demonstrating that the CD80–PD-L1 *cis* interaction inhibits the PD-1–PD-L1 *trans* interaction while maintaining the CD80–CD28 *trans* interaction^19,43^. A model of this interaction is shown in Supplementary Fig. 8. In agreement with the structural data, we demonstrated PD-L1-dependent CD28 costimulation using a single variant CD80 IgV domain. Thus, simultaneous binding of both PD-L1 and CD28 is not only possible, it is a functionally active interaction.

The clinical success of CTLA-4 and PD-1/PD-L1 CPI has illuminated the utility of harnessing the immune system for generating anti-tumor responses. However, despite the early success with monotherapy CPI, it is now clear that further improvements in rates and durability of responses may require combining checkpoint inhibition with novel therapies that stimulate a proinflammatory immune response within the tumor microenvironment^44^. Of the many T cell costimulatory receptors, CD28 is arguably the most potent, resulting in proinflammatory cytokine production, enhanced T cell survival and proliferation, and increased effector functions when combined with TCR signaling.

Recent reports suggest that both CTLA-4 and PD-1 preferentially suppress CD28 signaling within the tumor, leading to reduced T cell activation, effector function, and metabolism^9,10,15^. In addition to upregulation of CTLA-4 and PD-1 on tumor-infiltrated T cells, in many tumors, the CD28 ligands CD80 and CD86 are poorly expressed both on tumor cells and tumor-associated myeloid cells^11–13,45^. These observations have led several groups to generate novel therapeutics that provide CD28 costimulation either non-specifically, as with WT CD80 ECD-Fc^46^, or specifically via a tumor antigen (TA)/CD28 bispecific antibody^47^ or TA/CD3/CD28 trispecific antibody^48^. Our approach, however, takes advantage of CD80’s unique capacity to bind both PD-L1 and CD28 simultaneously, enabling ALPN-202 to elegantly combine dual checkpoint inhibition with PD-L1-dependent CD28 costimulation in a single therapeutic entity. We anticipate that this combination will generate more potent, clinically meaningful anti-tumor immunity than checkpoint inhibition alone.

## Methods

We affirm that all research described herein complies with all relevant human and animal ethical regulations.

### Directed evolution of human CD80

Yeast surface display libraries containing the randomly mutated IgV domain of human CD80 (residues 35-141) were generated by error-prone PCR as described^21^. Multiple selection strategies were employed to generate a diverse set of candidates. All intermediate selection outputs were assayed for CD28, CTLA-4, and PD-L1 binding by flow cytometry. The pool of mutants from which the lead candidate domain (H2393) was isolated and evolved as follows. A randomly mutated library (2.7 × 10^8^ clones, avg 4.6 mutations/clone) was screened with 2 parallel flow sorting progressions: (A) 3 nM CTLA-4-Fc^+^ (produced at Alpine Immune Sciences) followed by two successive 300 nM CD28-Fc^−^ (R&D Systems) sorts and (B) two successive 300 nM CD28-Fc^−^ sorts followed by a 3 nM CTLA-4-Fc^+^ sort. Yeast plasmid DNA isolated from the A and B terminal sorts was pooled and used as template to build a subsequent error-prone PCR library. This second-round library was screened via the following sort progression: 50 nM PD-L1-Fc^+^ (R&D Systems) followed by 20 mM CTLA-4-Fc^+^, followed by 10 nM PD-L1-Fc^+^ followed by 25 nM CD28-Fc. Each sort step was followed by outgrowth in YPD media (Himedia) at 30 °C and expression induction in media derived from Benatuil et al. (2010), with the substitution of dextrose/galactose with dextrose only at 22 °C before progressing to the next sort.

### Recombinant protein production and purification

CD80 vIgDs were expressed as soluble Fc-fusion proteins as described^21^. Recombinant vIgD Fc-fusion proteins and native IgSF receptor ECD Fc-fusion proteins were produced via transient expression in Expi293F^TM^ cells (Invitrogen Thermo Fisher Scientific) following the manufacturer’s protocol. Protein was purified from conditioned media harvests by capture and elution from Protein A (MabSelect SuRe, GE Healthcare) followed by a Size Exclusion Chromatography (SEC) polishing step (HiLoad 16/600 Superdex 200 pg, 1.6 × 60 cm, GE Healthcare) to generate monomeric, highly purified material. Protein concentrations were based on 280 nm absorbance using extinction coefficients calculated from the predicted primary structure^49^. Purified proteins were formulated in 10 mM acetate, 9% sucrose, pH 5.0, and stored at −80 °C. A sample vial was thawed and assessed by analytical SEC to demonstrate stability after thaw. Analytical SEC was performed on a G3000SW XL column (7.8 mm × 30 cm, 5 µM, Tosoh Biosciences) at a flow rate of 0.5 mL min^−1^ using an Alliance 2695 (Waters) with 10 mM Acetate, 250 mM NaCl, pH 5 as running buffer. Purified proteins (50 μl) were applied at 0.5 mg ml^−1^. Additionally, material was tested for endotoxin (Charles River Laboratories LAL endotoxin kit). Endotoxin levels for all proteins were <1 EU/mg.

### Crystallization of ALPN-202:PD-L1 complex

Monomeric ALPN-202 vIgD domain and PD-L1 ECD domain were prepared as carboxyl terminal His tagged recombinant proteins expressed in Expi293F^TM^ cells, purified by affinity chromatography on IMAC columns (HisTrap Excel, GE Health Care) and buffer exchanged into 10 mM acetate, 9% sucrose, pH 5.0. The ALPN-202:PD-L1 complex was prepared by mixing ALPN-202-derived CD80 vIgD His and PD-L1 ECD His proteins at a 1.2:1 molar ratio and the CD80 vIgD:PD-L1 complex was isolated by SEC fractionation. The complex was concentrated, flash frozen in liquid nitrogen and stored at −80 °C until ready for crystallization.

Crystals of the CD80 vIgD in complex with PD-L1 were grown using the vapor diffusion method. The complex crystals were grown at 6.7 mg mL^−1^ from reservoir solution (0.03 M sodium nitrate, 0.03 M sodium phosphate dibasic, 0.03 M ammonium sulfate, 0.1 M sodium HEPES/MOPS (acid), pH 7.5, 20% v/v glycerol, 10% w/v PEG 4000). Crystals were frozen directly from the crystallization drop.

Datasets were collected at 100 K at station I03, Diamond Light Source, Didcot, England (λ = 0.9763 Å) equipped with a Pilatus3 6 M detector. Data were processed using XDS^50^ and merged using Aimless^51^. A data set for ALPN-202 CD80 vIgD in complex with PD-L1 was collected to 3.15 Å. Crystals belonged to space group P2_1_2_1_2_1_ and had cell dimensions: *a* = 59.9, *b* = 122.2, *c* = 152.7 Å; α, β, and γ = 90°.

The structure was determined by molecular replacement using the Molrep software^52^. Published CD80 (PDB: 1DR9^30^) and PD-L1 (PDB: 5JDR^53^) structures were used as initial models and the binding surface and contact residues within 4 Å between the CD80 vIgD and the PD-L1 ECD were determined by using CONTACT of the CCP4 software package^54^. Two complexes of ALPN-202 and PD-L1 were found in the asymmetric unit. The structure was refined using Refmac5^55^, then in Buster (Global Phasing Ltd.), and model building was carried out in Coot^56^. In the final refinement, the crystal structure of PD-L1 to 1.95 Å resolution (PDB: 4Z18), was used as a target for geometrical restraints. The structure of the CD80 vIgD in complex with PD-L1 includes amino acids 35–140 of the CD80 vIgD in chain A, 19–227 of PD-L1 in chain B, 35–57, 61–77, 80–122, and 126–140 of the CD80 vIgD in chain C, and 19–227 of PD-L1 in chain D. The side chains of Asn-53 and Asn-89 of CD80 vIgD (chains A and C), and Asn-35, Asn-192 and Asn-200 of PD-L1 were glycosylated. Thr-127 of PD-L1 in chain B was phosphorylated. Ramachandran plot quality was calculated using MolProbity v4.02b-467^57^. The data and model quality parameters are in Supplementary Table 2. The average area covered by the binding interactions was calculated using AREAIMOL (Supplementary Table 3). AIMLESS, Molrep, Coot, AREAIMOL, and CONTACT are all part of the CCP4 Software Suite v7.0.044 of the CCP4 software package.

### Accession number

Structure coordinates of the ALPN-202 CD80 vIgD:PD-L1 ECD complex have been deposited in the worldwide Protein DataBase (wwPDB) with accession number 7TPS.

### Modeling of trimeric CD28:ALPN-202:PD-L1 complex

The ALPN-202 CD80 vIgD:PD-L1 ECD structure was co-aligned with both the B7-1:CTLA-4 crystal structure (PDB: 1I8L^32^) and chain C from the CD28-Fab crystal structure (PDB: 1YJD^51^) to demonstrate availability of co-binding between the molecules. The ALPN-202 CD80 vIgD:PD-L1 structure was aligned with the PD-1:PD-L1 crystal structure (PDB: 4ZQK^58^) to demonstrate the blocking potential of ALPN-202 CD80 vIgD to the binding of PD-1 by PD-L1. Structure modeling was performed using The PyMol Molecular Graphics System Version 2.3.3 (Schrödinger, LLC).

### Generation of stably transduced cell lines

Mammalian cells expressing cell surface CD28, CTLA-4, and PD-L1 were generated independently by cloning DNA encoding the full-length proteins into the lentiviral vector pRRL downstream of an MND promoter and Kozak sequence located downstream of the cPPT element. All inserts were preceded by native signal peptides to direct cell surface expression. To monitor lentiviral transduction efficiency and selection of stable integrants, protein expression was translationally coupled to EGFP or a puromycin resistance gene via an intervening T2A peptide^59^. Lentiviral vectors, and helper plasmids pMD2.G and psPAX2 were transfected into Expi293F^TM^ (Thermo Fisher Scientific) cells to generate lentiviral particles. Viral particles were used to infect target cells for stable lentiviral expression^60^. For some experiments, the pLVX-EF1α-IRES-Puro lentiviral vector, Lenti-X Packaging Single Shots packaging plasmids and Lenti-X 293 X packaging cell line were used to generate viral particles per the manufacturer’s protocol (Clontech, Takara Bio). Viral supernatants were collected 48–72 h after transfection and in some cases concentrated using the Lenti-X^TM^ Concentration kit (Clontech, Takara Bio) per the manufacturer’s protocol. Target cells were seeded in growth media in a 6-well plate (Corning) and 1 mL virus was added with Polybrene at a final concentration of 10 μg mL^−1^. The plate was spun at 2500 rpm for 30 min and incubated overnight at 37 °C in 5% CO_2_. Media was changed the next day and protein expression was monitored by flow cytometry.

### Cell lines and primary cells

GloResponse™ IL-2-*luc2P* Jurkat cells (Promega) were cultured in RPMI 1640 supplemented with 10% fetal bovine serum, 1 mM sodium pyruvate and 200 µg mL^−1^ hygromycin B. aAPCs were K562 cells (ATCC #CCL-243) virally transduced to stably express a transmembrane anti-CD3 single chain Fv (scFv) and/or wild type PD-L1. Expi293™ cells (Thermo Fisher Scientific) were used to produce proteins or transiently express mouse CD28, CTLA-4, and PD-L1. ExpiCHO-S™ cells (Thermo Fisher Scientific) were virally transduced and selected for stable expression of human CD28, PD-L1, or CTLA-4. MC38 (originally from the laboratory of Dr James Allison), B16-F10 (ATCC #CRL-6475), and SCC152 (ATCC #CRL-3240) cells were virally transduced and selected under puromycin (2%) for stable expression of human PD-L1. CD28, PD-L1, or CTLA-4 expression was confirmed by flow cytometry.

Human whole blood as well as cryopreserved peripheral blood mononuclear cells and purified T cells were purchased from Bloodworks Northwest, an AABB and CLIA accredited agency. Monocytes were isolated from LRS chambers (Bloodworks Northwest) using the EasySep™ Human Monocyte Isolation Kit (StemCell Technologies). Blood and blood products (cells) were collected from heathly, consented adults under WIRB Protocol #20151321 (NML DONOR_BLOOD_002).

### Binding affinity measurement by surface plasmon resonance (SPR)

Affinity determination was conducted on a Biacore 3000 (GE) optical biosensor equipped with a CM5 sensor chip prepared with goat anti-human IgG capture antibody (Jackson ImmunoResearch Laboratories). ALPN-202 or WT CD80 ECD-Fc was captured to a level of 120 RU and 160 RU, respectively. For kinetic assays, serial dilutions of recombinant monomeric human CD28, CTLA-4, and PD-L1 (Acro Biosystems) were prepared as follows: CD28, 500 nM to 6.17 nM; CTLA-4, 175 nM to 2.16 nM; and PD-L1, 1500 nM to 18.5 nM. Each analyte titration was run in triplicate in two separate experiments and multiple blank (buffer) injections were conducted to assess and subtract system artifacts. Association phases for all analyte concentrations were monitored for 240 s, while the dissociation phases were collected for 600 s, at a flow rate of 30 μL/min.

Data were aligned, double referenced, and fit using Scrubber v2.0^®^ software (BioLogic Software Pty Ltd.). For kinetic data, association and dissociation phases were globally fit to the 1:1 binding model, to determine ka, kd, and Rmax values. KD was determined by the quotient of the dissociation rate to the association rate. For the weak CD28–WT CD80 and PD-L1–WT CD80 interactions, KD was estimated and expressed as a minimal KD by using the theoretical Rmax as a fixed parameter in the global fit.

### Binding to human and mouse CTLA-4, CD28, and PD-L1 by flow cytometry

ExpiCHO cells stably expressing human or mouse CTLA-4, CD28 or PD-L1 were cultured in shake flasks containing ExpiCHO™ Expression Medium (Thermo Fisher Scientific) in a 37 °C, 8% CO_2_ humidified incubator. To measure binding to CD80 counter-structures, CHO cells were plated in a 96-well U-bottom plate and incubated 60 min on ice with serial dilutions of ALPN-202, WT hCD80-Fc, WT mCD80-Fc, or Fc control. Cells were washed and bound drug detected using PE or Alexa Fluor® 647-conjugated polyclonal anti-human Fc (Jackson ImmunoResearch). Cells were analyzed using a Becton Dickinson LSRII flow cytometer and data analyzed using FlowJo v7 software.

### Blockade of PD-1–PD-L1, CD28–CD80 and CTLA-4–CD80

CD80-Fc and PD-1-Fc proteins (R&D Systems) were conjugated with Alexa Fluor^®^ 647 (Molecular Probes^®^ Alexa Fluor^®^ Antibody Labeling Kit, Thermo Fisher Scientific) according to manufacturer’s instructions. To measure blockade of PD-1 - PD-L1, K562/PD-L1 cells were plated in a 96-well plate and incubated with serial dilutions of ALPN-202, anti-PD-L1 (atezolizumab), or Fc control. Cells were washed and incubated with PD-1-Alexa Fluor 647. Bound PD-1 was detected using a Becton Dickinson LSRII flow cytometer and data analyzed using FlowJo v7 software. To measure blockade of CD28 - CD80 and CTLA-4 - CD80, CHO cells stably expressing CD28 or CTLA-4 were plated in 96-well plates and incubated with serial dilutions of ALPN-202, anti-CD28 (clone 28.2, BioLegend), anti-CTLA-4 (ipilimumab) or Fc control. Cells were washed and incubated with CD80-Alexa Fluor 647. Bound CD80 was detected using a Becton Dickinson LSR II flow cytometer and data analyzed using FlowJo v7 software.

### SHP-2 recruitment assay

40,000 PathHunter^®^ Jurkat PD-1/SHP-2 cells (DiscoverX) were co-cultured with 60,000 K562/PD-L1 cells in the presence of ALPN-202, WT CD80 ECD-Fc, anti-PD-1 (nivolumab), anti-PD-L1 (atezolizumab), or Fc control. Test articles were serially diluted and added to cell plate for final concentrations of 200 nM to 0.003 nM. After 2 h, detection reagent (DiscoverX) was added to each well and luminescence measured.

### Artificial APC T cell stimulation assay

K562 cells stably expressing membrane-anchored anti-CD3 single chain Fv (clone OKT3) and/or PD-L1 were plated at 12,500 cells/well in 96-well plate. 1 × 10^5^ primary T cells (Bloodworks Northwest) and serial dilutions of each test article were added to each well. In some cases, titrations of ALPN-202 or monomeric CD80 vIgD were combined with 100 nM blocking anti-PD-L1 or anti-CD28. Plates were incubated 24 h at 37 °C. Secreted IL-2 was measured using a LEGEND MAX™ Human IL-2 ELISA kit (BioLegend).

In separate experiments, 1 × 10^5^ primary T cells were co-cultured with 2 × 10^4^ K562/PD-L1 cells and incubated with a 100 nM–0.02 nM ALPN-202, agonist anti-CD28 (clone 28.2, BioLegend), Fc control, anti-PD-L1 (atezolizumab), anti-CTLA-4 (ipilimumab), or a combination of anti-PD-L1 and anti-CTLA-4. Submaximal TCR stimulation was provided by 12.5, 2.5, 0.5, or 0 nM anti-CD3 (clone OKT3, BioLegend). After 16 h supernatants were collected and analyzed for secreted IL-2. Cells were then treated with brefeldin A/monensin for 6 h and characterized by flow cytometry for intracellular IL-2 and CD25 upregulation. Four donors were tested in two separate experiments with all samples run in triplicate.

### Simultaneous CD28 and PD-L1 binding to ALPN-202 by SPR

ALPN-202 was captured to the sensor chip as described previously and monomeric analytes (1500 nM CD28, PD-L1, or CTLA-4) were sequentially co-injected to saturate binding with a 180 s association phase for each injection at a flow rate of 30 μL min^−1^. Single analyte injections were paired with blank buffer injections as controls. Samples were run in duplicate and sensorgram overlays depicted in Fig. 2e.

### Generation of human E6 TCR transgenic T cells

Cryo-preserved purified primary human T cells (Bloodworks Northwest) were thawed and incubated 6 h in media containing 100 IU/mL IL-2 and anti-CD3/anti-CD28 beads (Dynal) at a 2:1 bead to cell ratio. Titered viral stocks were added to attain a multiplicity of infection of 4 and incubated overnight at 37 °C. Viral supernatants were removed and fresh media including 100 IU/mL IL-2 was added. Cultures were incubated 2 days at 37 °C. On day 3, beads were removed by magnetic selection and cells were replated in media containing IL-2 as above. Cultures were incubated for an additional 7 days with media changes every two days including IL-2 as above. T cells were harvested on day 10 and transduction efficiency was evaluated by flow cytometry for the presence of the mouse Cβ constant region included in the E6 TCR.

### T cell and M2c macrophage coculture assays

M2c macrophage polarization was performed in 96-well plates by differentiating 50,000 monocytes/well in complete X-Vivo 15 media (Lonza) with 100 ng/mL of M-CSF for 5 days. Differentiated macrophages were polarized to an M2c phenotype with 50 ng/mL of IL-10 for 2 days. Alternatively, differentiated macrophages were polarized to an M1 phenotype with 100 ng/mL of IFNγ and 1 ng/mL of LPS for a positive control. Macrophages were characterized by flow cytometry using 1:100 dilutions of antibodies specific for human CD80, CD86, CD163, CD206, MHC class II, and PD-L1 (Supplementary Fig. 5). (Fig. 4a–d) Polarized macrophages in each well were co-cultured with 5 × 10^5^ autologous CellTrace™ Violet (CTV; Thermo Fisher Scientific)-labeled T cells with different concentrations of test articles and 100 ng/mL anti-CD3 (clone OKT3). Cell culture supernatants were harvested at 24 h for IL-2 quantitation by HTRF (Cisbio). Dilution of CTV was used to measure T cell proliferation at 72 h. Supernatants were also harvested at end of assay to measure IFNγ, IL-2, TNFα, GM-CSF and IL-21 using a multiplex bead-based magnetic MAGPIX™ assay (Luminex). (Fig. 4e) M2c polarized macrophages, E6 TCR^+^ T cells, and PD-L1^+^ SCC-152 tumor cells (25,000 each) were co-cultured in triplicate wells for 24 h in the presence of test articles. Supernatants were collected and soluble IFNγ, IL-2, and TNFα were quantitated.

### Jurkat IL-2 reporter assay

K562/PD-L1 cells expressing membrane-anchored anti-CD3 scFv (clone OKT3) were plated at 25,000 cells/well in a white, 96-well plate. 1 × 10^5^ GloResponse™ IL-2-*luc2P* Jurkat cells (Promega), serial dilution of monomeric CD80 vIgD, and 100 nM anti-CD28, anti-PD-L1 or Fc control was added to each well. Plates were incubated 5 h at 37 °C. After incubation, an equal volume of BioGlo™ Assay Reagent (Promega) was added to each well and luminescence measured.

### Mouse tumor studies

All mouse studies were performed under Alpine Immune Sciences (AIS)-affiliated Institutional Animal Care and Use Committee (IACUC) protocol number 5305-01. During the development and evaluation of therapeutics in mouse tumor models (including the human PD-L1 transduced MC38, B16-F10, and SCC152 models in this paper), we conducted power calculations to estimate minimum sample sizes that are appropriate for determining statistically significant differences in tumor volumes over time (power = 0.80; alpha = 0.05). The results of those calculations (9–10 animals per group for tumor studies) are used for all studies in which the same model and Fc control treatment are used. For all MC38 and B16-F10 tumor studies, female C57BL/6NJ mice (8–10 weeks old at study start) were purchased from the Jackson Laboratory. For the SCC152/hPD-L1 tumor model using human E6 TCR Tg T cells, female NOD-*scid* IL2Rgamma^null^ (NSG) mice (aged 7–8 weeks) were purchased from Jackson Laboratory. All mice were housed under specific pathogen-free conditions at 24 ± 2 °C on a 12-h light/12-h dark cycle at the Association for Assessment and Accreditation of Laboratory Animal Care (AAALAC)-approved animal facility affiliated with AIS. All mice had free access to food and water at all times. Tumor progression was monitored twice weekly by caliper measurements and tumor volume was calculated as length × (width^2^)/2. Though maximum allowable tumor volume was 2000 mm^3^, mice were euthanized when tumor volumes reached 1500 mm^3^ or appeared ulcerated. No tumors reached the maximum 2000 mm^3^. Data were analyzed and presented as mean tumor growth ± SEM. Statistical significance was determined using repeated measures two-way ANOVA.

### MC38 and MC38/hPD-L1 tumor studies

1.5 × 10^6^ MC38 cells were implanted subcutaneously (SC) into the flanks of C57BL/6NJ mice. After 7 days (mean tumor volumes were 53–104 mm^3^), mice were randomly assigned to treatment groups and treatments initiated either the same day or the following day via intraperitoneal (IP) administration. For repeat-dose studies comparing ALPN-202 to WT CD80 ECD-Fc, anti-hPD-L1 (durvalumab; Catalent), anti-mCTLA-4 (clone 9D9; BioXcell), anti-mPD-1 (clone RMP1-14; BioXcell) or anti-mCD28 (clone E18; mouse IgG2b Fc silent^™^; Absolute Antibody), mice were treated with one or three doses (depending on the study) of 100 μg of ALPN-202, 100 μg of the appropriate antibody (anti-hPD-L1, anti-mCTLA-4, anti-mPD-1, or anti-mCD28 as noted above), 130 μg of WT CD80 ECD-Fc or 75 μg of Fc control every 2 or 3 days as indicated. For the dose-ranging study (Fig. 5f, g, and Extended Data Fig. 6), single IP injections of 20, 100, 500, or 1500 μg ALPN-202 or 75 μg of Fc control were administered on day 8. Serum was collected from subsets of 3 mice per group per time point at various time points after dosing (1–840 h) for pharmacokinetic (PK) analysis. Serum concentrations of ALPN-202 were determined by an ELISA developed at AIS and PK parameters estimated using Phoenix WinNonlin v6.4 software (Certara). In studies that included ex vivo analysis of tumors by flow cytometry (Figs. 5, 6) or RNA-Seq (Fig. 5), mice were randomized to the ex vivo subgroups at the time of staging on day 7. The ex vivo subgroups had similar mean tumor volumes as the mice used to monitor tumor growth. To evaluate the effect of blocking PD-L1 or CD28 antibodies on ALPN-202 responses in vivo, ALPN-202 was co-administered with either anti-hPD-L1 (durvalumab) or an anti-mCD28 blocking antibody (clone E18) administered as 100 μg IP injections on days 8, 11, and 14.

### B16-F10 tumor studies

Female C57BL/6NJ mice were implanted SC with 0.5 × 10^6^ B16-F10/hPD-L1 cells into the right flank on Day 0 and sorted into groups of 13. On days 6, 8, and 11, mice received IP injections of 75 μg Fc control, 100 μg ALPN-202, 100 μg anti-mPD-1 (clone RMP1-14; rat IgG2a; BioXcell), or a combination of ALPN-202 and anti-mPD-1.

### SCC152 xenograft tumor studies

Female NOD-*scid* IL2Rgamma^null^ (NSG) mice (aged 7–8 weeks) were SC implanted in the right flank with 4 × 10^6^ SCC152/hPD-L1 cells prepared in Matrigel (Corning). On day 12, when tumors were 80–120 mm^3^, animals were SC dosed with 10 mg hIgG to reduce FcR-mediated effects of therapeutic treatments. On day 14, animals were randomized into groups of 10 mice each. On day 15, 5 × 10^6^ human E6 TCR^+^ T cells/mouse were transferred via retro-orbital injection. On day 16, test articles were administered as follows via IP dosing: Fc control (75 μg/dose), every 3 days for 4 doses (Q3Dx4); ALPN-202 (100 μg/dose), Q3Dx4; ALPN-202, every 7 days for 3 doses (Q7Dx3); anti-hPD-L1 (durvalumab) (100 μg/dose), Q7Dx3.

### Flow cytometry of MC38 tumors ex vivo

MC38 tumors from 5 mice/group were harvested either 72 h after a single injection (Fig. 5g) or 48 h after the second injection of indicated treatments (Fig. 6c–e). Tumors were enzymatically digested at 37 °C for 42 min using a Mouse Tumor Digestion Kit (Miltenyi Biotec) and a gentleMACS™ Octo Dissociator with Heaters (Miltenyi Biotec) according to manufacturer’s instructions for soft and medium tumors. For each tumor 0.5–1 × 10^6^ live cells were stained with a viability stain (LIVE/DEAD Fixable Aqua, Life Technologies Corp.) according to manufacturer’s instructions, followed by resuspension of the tumor pellets in 5 μg mL^−1^ mouse Fc block (clone 2.4G2; BD Biosciences) and a 45 min incubation on ice with a cocktail of fluorescently-labeled anti-mouse antibodies against CD45 (1:100 dilution; clone 30-F11; BioLegend), CD3ε (1:50 dilution; clone REA606; Miltenyi Biotec), CD4 (1:100 dilution; clone GK1.4; BioLegend), CD8α (1:100 dilution; clone KT15; Invitrogen), and CD11b (1:100 dilution; clone M1/70; BioLegend). In the study that included a PE-conjugated mouse class I p15E tetramer to identify antigen-specific CD8^+^ T cells (Fig. 5g), 7.5 μl tetramer (1:13 dilution; MBL International) was pre-incubated with each tumor cell suspension sample for 30 min at 25 °C prior to addition of the flow antibody cocktail. For granzyme B intracellular staining, surface-stained cells were fixed and permeabilized using an intracellular fix/perm buffer set (BioLegend) prior to a 45 min incubation at 25 °C with anti-mouse granzyme B (1:20 dilution; clone QA16A02; BioLegend). Stained samples were washed and collected onto a Becton Dickinson LSRII flow cytometer. Cell subsets were identified and quantified using FlowJo v10 software using the gating scheme shown in Extended Data Fig. 7.

### RNA-Seq analysis of MC38 tumors ex vivo

Mice implanted with MC38/hPD-L1 cells were allowed to reach tumor volumes of 60 mm^3^ (~7 days). Mice were randomized and treated with a single IP injection of control Fc protein (75 µg), ALPN-202 (100 µg), or anti-PD-L1 (durvalumab) (100 µg) on day 8. Four tumors per treatment group were harvested after 72 h and RNA was prepared from tumor sample lysates.

Total RNA (0.5 ng) was added to lysis buffer from the SMART-Seq v4 Ultra Low Input RNA Kit for Sequencing (Takara), and RT-PCR to generate full-length amplified cDNA was performed. Sequencing libraries were constructed using the NexteraXT DNA sample preparation kit (Illumina) to generate Illumina-compatible barcoded libraries. Libraries were pooled and quantified using a Qubit® Fluorometer (Life Technologies). Dual-index, single-read sequencing of pooled libraries was carried out on a HiSeq2500 sequencer (Illumina) with 58-base reads, using HiSeq v4 Cluster and SBS kits (Illumina) with a target depth of 5 million reads/sample. Base calls were processed to FASTQs on BaseSpace (Illumina), and a base call quality-trimming step was applied to remove low-confidence base calls from the ends of reads. The FASTQs were aligned to the mouse reference genome, using STAR v.2.4.2a and gene counts were generated using htseq-count. QC and metrics analysis was performed using the Picard family of tools (v1.134).

Gene Transcripts Per Million (TPM) were calculated using the gene lengths from NCBIM37 as downloaded via BioMart (ensembl.org) for the May 2012 version. The counts for each gene were divided by gene length and multiplied by 1000. Then all values were divided by the number of million reads in that sample.

Evaluation of differential gene expression between ALPN-202 and Fc control treated tumors was performed using the R-based Needle Genomics Expression Atlas (Needle Genomics). Gene signature analysis was performed by identifying the 200 most differentially regulated genes comparing ALPN-202 with Fc-control treated tumors using false discovery rate (FDR) and focusing on genes upregulated >2x, which yielded a list of 124 genes. Signature transcripts from the indicated immune cell populations were identified using the ImmGen Population Comparison application [https://www.immgen.org/ImmGenpubs.html]. Pathway analysis with this gene set was carried out using the online Enrichr Pathway Analysis tool [https://maayanlab.cloud/Enrichr/].

### Cytokine release assay

For soluble assay, human PBMC from three separate donors (Bloodworks Northwest) were thawed in X-VIVO 15™ media (Lonza) supplemented with antibiotics (100 U mL^−1^ penicillin, 100 µg mL^−1^ streptomycin) and GlutaMAX™ (Thermo Fisher Scientific) and plated at 1.0 × 10^6^ cells/well in quadruplicate in a round-bottom 96-well plate. Test articles were added to appropriate wells incubated for 24 h at 37 °C prior to collection of supernatants. For the solid phase assay format, test proteins were diluted in PBS and aliquoted into 96-well round-bottom plates and incubated overnight at 4 °C. The next day, PBMC were thawed as described above and rested for 3 h at 37 °C at high density. Rested PBMC were added to assay plates and incubated for 24 h at 37 °C. Supernatants were assayed for secreted IFNγ, IL-2, IL-4, IL-6, IL-8, IL-10, and TNFα using a custom MILLIPLEX™ MAP Human Magnetic Bead array kit (Millipore) and following the manufacturer’s instructions. Data were collected on the Millipore MAGPIX™ System.

### Statistics and reproducibility

For in vitro assays, mean or median ± SD or SEM was calculated using GraphPad Prism™ v8. Experiments were independently repeated two or three times and representative data shown. Between three and five donors were used for in vitro assays using human cells to account for donor-to-donor variability. No data were excluded from the analyses.

We conducted power calculations to estimate minimum sample sizes appropriate for determining statistically significant differences in tumor volumes over time (power = 0.80; alpha = 0.05). The results of those calculations (9-10 animals per group for tumor studies) are used for all studies in which the same model and Fc control treatment are used. While not officially blinded, tumor volumes were measured by caliper in a predetermined, consistent manner by individuals with no/limited knowledge of the function of the test articles. Tumor growth curves over time were evaluated for significant differences between treatment groups by repeated-measures two-way ANOVA for ‘treatment’ effects. Ex vivo tumor flow cytometry and RNA transcript data were analyzed by one-way ANOVA with Dunnett’s multiple comparisons test versus Fc control or ALPN-202 (Figs. 5–7). Significant differences between groups for all statistical analyses (*P* < 0.05) were calculated using GraphPad Prism 8. For Enrichr Pathway Analyses, *P*-values were calculated using Fisher’s exact test. The adjusted *P*-value was calculated using the Benjamini-Hochberg method for correction of multiple hypotheses testing. The Odds Ratio was computed using a modification to Fisher’s exact test to indicate a risk of deviation from the expected rank. Combined score was calculated by multiplying the ln(*P*-value) by the inverse of the Odds Ratio.

### Reporting summary

Further information on research design is available in the Nature Research Reporting Summary linked to this article.

## Supplementary information

Supplementary Information

Reporting Summary

Description of Additional Supplementary Files

Supplementary Data 1

## Data Availability

Structure coordinates of the ALPN-202 CD80 vIgD:PD-L1 ECD complex have been deposited in the worldwide Protein Data Bank (wwPDB; http://www.wwpdb/org) and been assigned accession number 7TPS. RNAseq data is available at the Gene Expression Omnibus (GEO) with accession number GSE161244. Source data for Figs. 1–7, and Supplementary Figs. 1–6 are provided with this paper as a Source Data file. The remaining data are available within the Article, Supplementary Information or Source Data file. Source data are provided with this paper.

## Source data

Source Data
