# Supplementary material for: The engineered CD80 variant fusion therapeutic davoceticept combines checkpoint antagonism with conditional CD28 costimulation for anti-tumor immunity
Source: Nat Commun. 2022 Apr 4;13:1790. doi: 10.1038/s41467-022-29286-5 (PMC8980021; doi:10.1038/s41467-022-29286-5)
Supplement: Supplementary file 1 — Supplementary Information [file 41467_2022_29286_MOESM1_ESM.pdf]

Supplementary Information

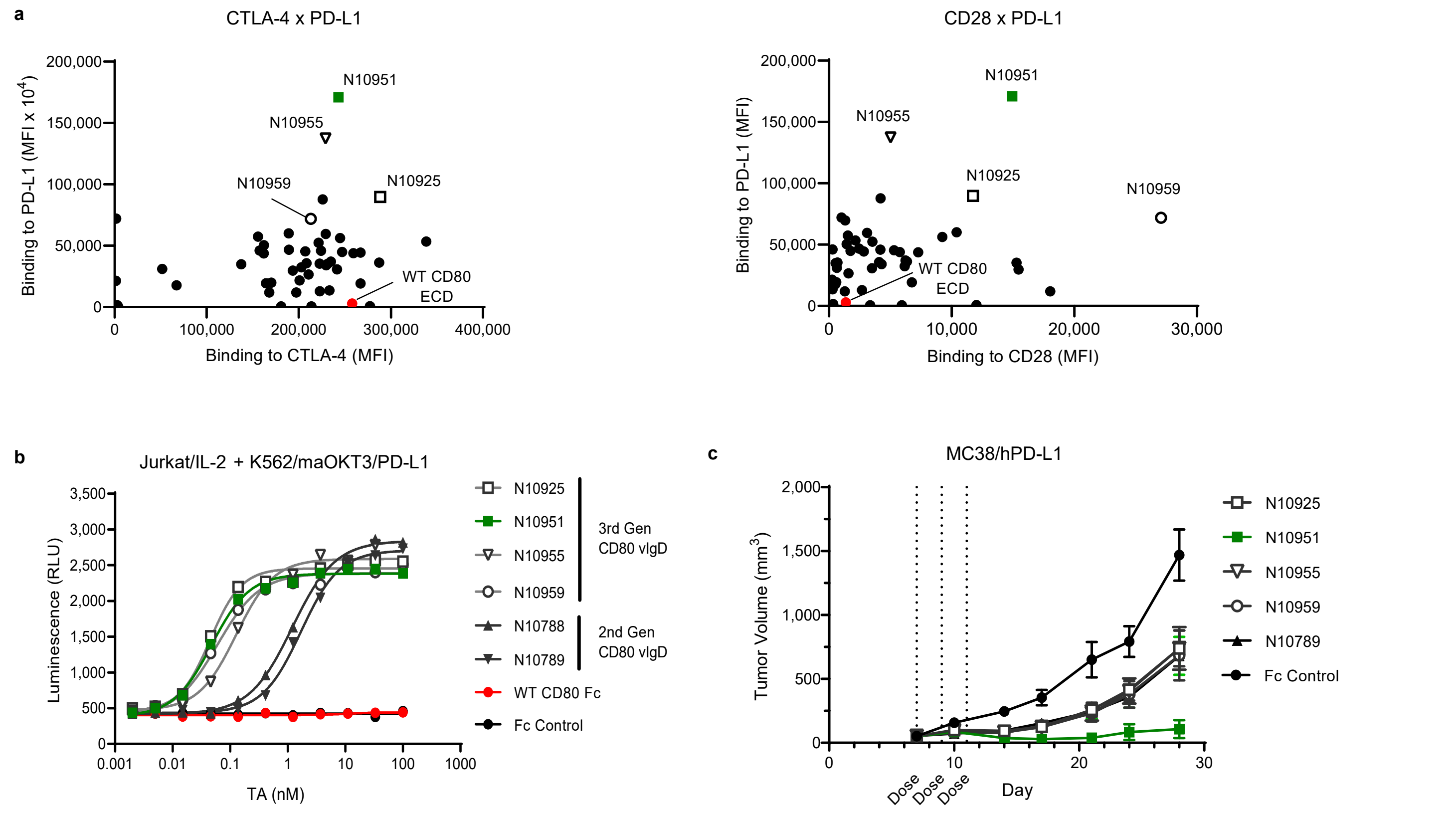

Supplementary Fig. 1 | ALPN-202 (N10951) was identified through multiple rounds of mutagenesis, binding screens and *in vitro* and *in vivo* functional testing. Sorting for high affinity PD-L1 binders led to a panel of variant CD80 IgV domain sequences (CD80 vIgDs). **a**, CD80 vIgDs were formatted and expressed as Fc dimers and purified proteins screened for binding to cells expressing human CD28, CTLA-4, and PD-L1. Each dot represents a different CD80 vIgD. **b**, CD80 vIgDs with high CD28 and PD-L1 affinity were screened in a Jurkat/IL-2 reporter assay designed to measure PD-L1-dependent CD28 costimulation. Data are representative to two independent assays. **c**. A subset of CD80 vIgDs was then tested in an MC38/hPD-L1 tumor model (n=11 mice/group; mean tumor volume ± SEM shown). N10951 was identified as having high affinity for human PD-L1, strong PD-L1-dependent CD28 costimulatory activity, and potent anti-tumor activity *in vivo,* and was renamed ALPN-202. Source data are provided as a Source Data file.

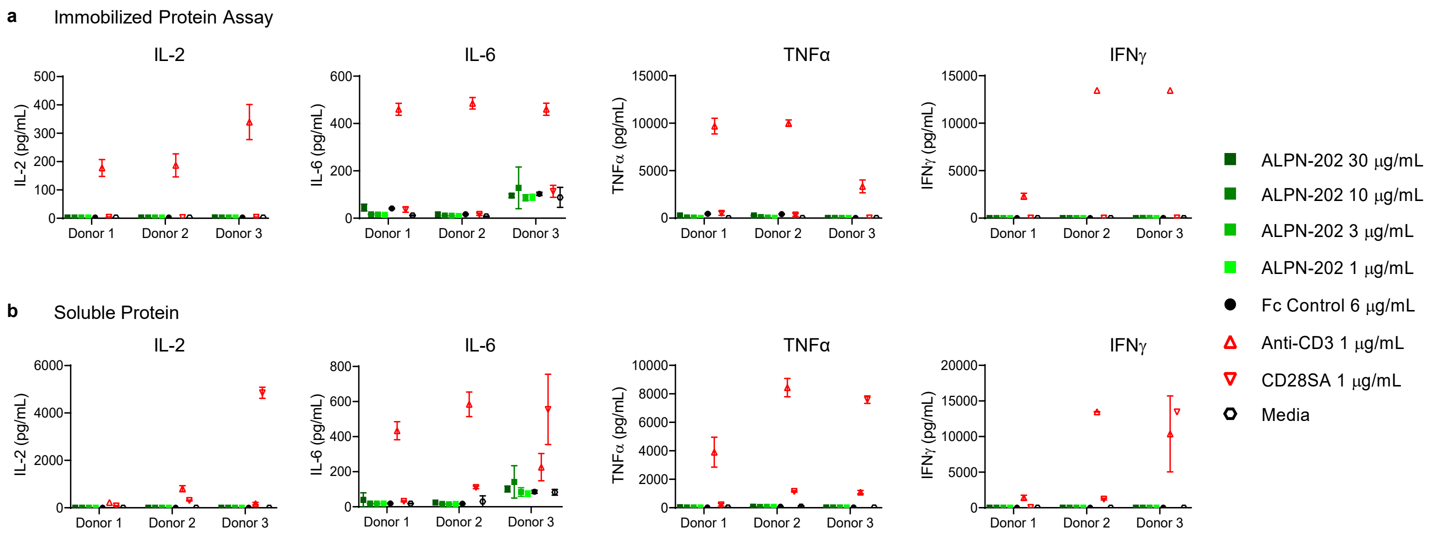

Supplementary Fig. 2 ALPN-202 does not activate T cells in absence of TCR activation. To determine if ALPN-202 induces cytokine production (i.e. superagonism) in the absence of TCR stimulation, high density PBMC co-cultures were established using either immobilized (**a**) or soluble (**b**) protein. ALPN-202 did not induce IL-2, IL-6, TNFα, or IFNγ production at any of the concentrations tested (n=3 donors). Each point shown is the mean concentration ± SD for each sample run in triplicate wells. Source data are provided as a Source Data file.

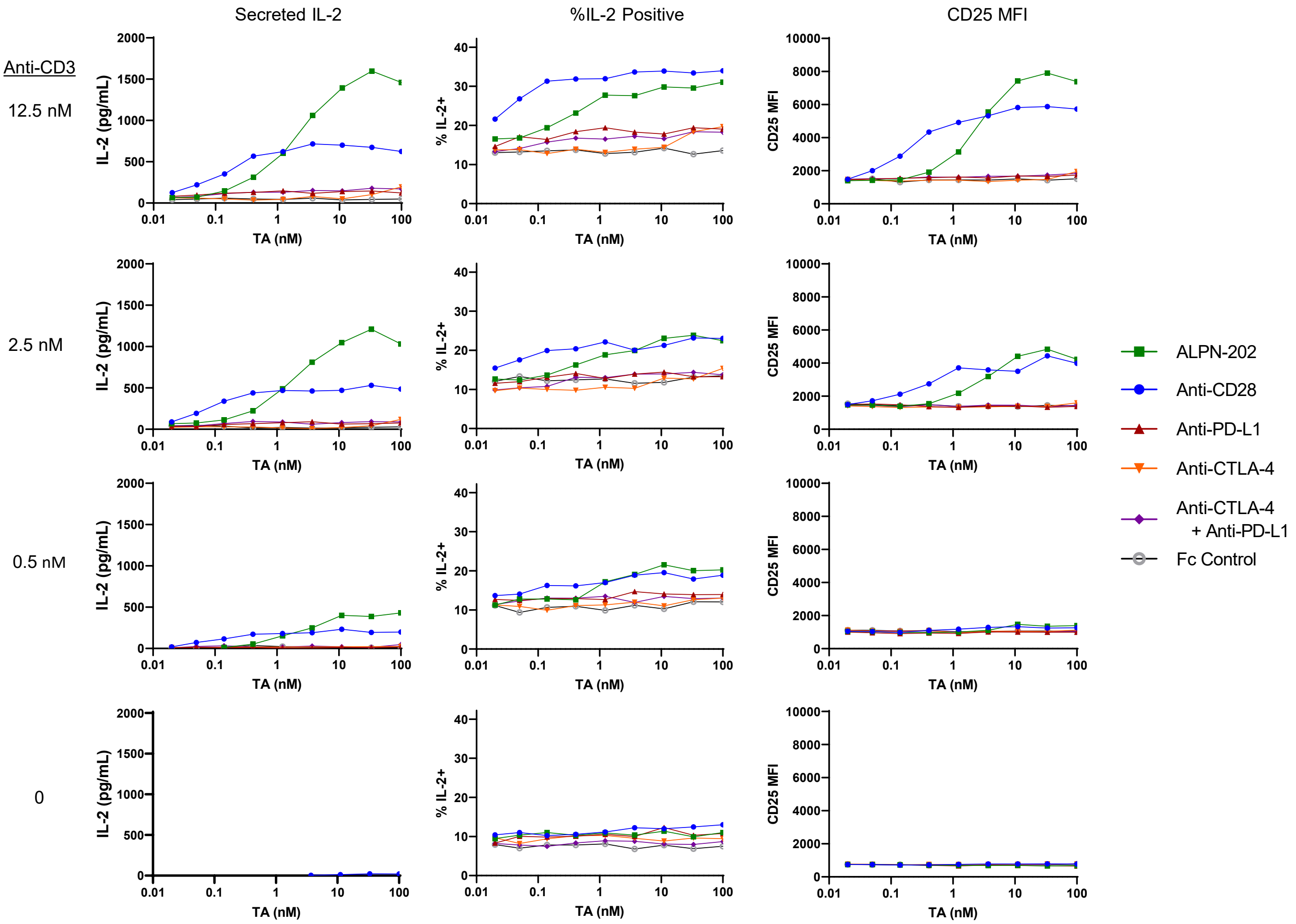

Supplementary Fig. 3 | ALPN-202 activity on T cells is TCR-dependent and is consistent with CD28 agonism. Human T cells were co-cultured for 24 hours with K562 cells and a titration of anti-CD3 antibody. Secreted IL-2 was measured at 16 hours and intracellular IL-2 and cell-surface CD25 median fluorescence intensity (MFI) was measured at 24 hours. ALPN-202 activity was CD3 stimulation-dependent, consistent with anti-CD28 antibody-mediated co-stimulation and clearly differentiated from single and dual CTLA-4 and PD-L1 inhibition. Representative data from one of three donors tested is shown. Secreted IL-2, %IL-2^{+}, and CD25 MFI values are mean values of duplicate wells. TA – test article. Source data are provided as a Source Data file.

Supplementary Table 1 | Contact residues in the ALPN-202 CD80 vIgD/PD-L1 ECD structure with atoms within 4.0 Å distance.

	Interaction residues
CD80 (chain A)	Lys 43, Glu 44, Val 45, Lys 88, Asn89, Arg 90, Thr 91, Ile 92, Asp94, Met 102, Leu104, Gly 105, Arg 107
PD-L1(chain B)	Ile 54, Tyr 56, Gln 66, Val 68, His 69, Glu 71, Arg 113, Ile116, Ser 117, Gly 120, Ala121, Asp122, Tyr 123
CD80 (chain C)	Lys 43, Glu 44, Val 45, Lys 88, Asn89, Arg 90, Thr 91, Ile 92, Asp94, Leu 104, Gly105, Arg 107
PD-L1(chain D)	Ile 54, Tyr 56, Gln 66, Val 68, His 69, Glu 71, Met115, Ile116, Ser 117, Gly 120, Ala121, Asp122, Tyr 123

a

Amino acid substitutions found in ALPN-202

	AA Position						
Protein	18	26	35	47	68	71	90
WT CD80 IgV	H	A	E	M	V	A	D
ALPN-202 Mutations	Y	E	D	L	M	G	G

b

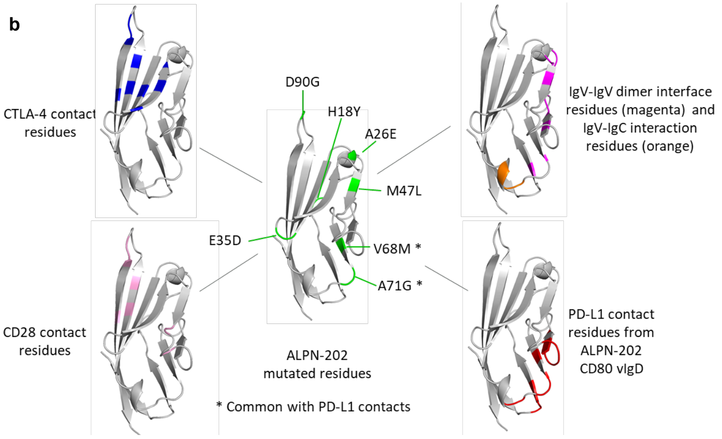

c

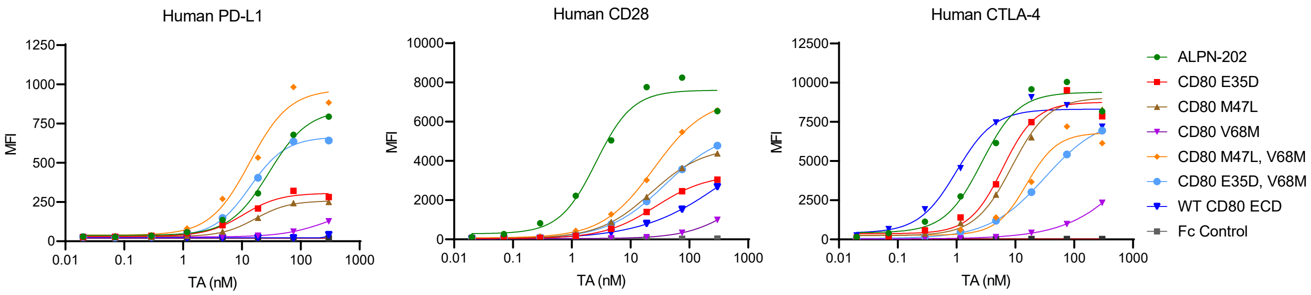

Supplementary Fig. 4 | ALPN-202 mutations, location, and impact relative to wild type CD80. **a**, Table summarizing the amino acid substitutions in ALPN-202 relative to the wild type CD80 IgV domain (WT CD80 IgV). **b**, The ALPN-202 CD80 vIgD structure is shown in grey with the location of the amino acid substitutions indicated in green. CD80 IgV-IgV and IgV-IgC contact residues within the CD80 homodimer are shown in magenta and orange respectively. The CD80 contact residues with CTLA-4 and CD28 are shown in blue and pink respectively. The ALPN-202 CD80 vIgD contact residues with PD-L1 are shown in red. **c**. Single and double mutation CD80 vIgDs were generated and evaluated for binding to cells expressing human PD-L1, CD28, and CTLA-4. Binding experiments were run two times with representative data shown. WT – wild type; ECD – extracellular domain. Source data are provided as a Source Data file.

a

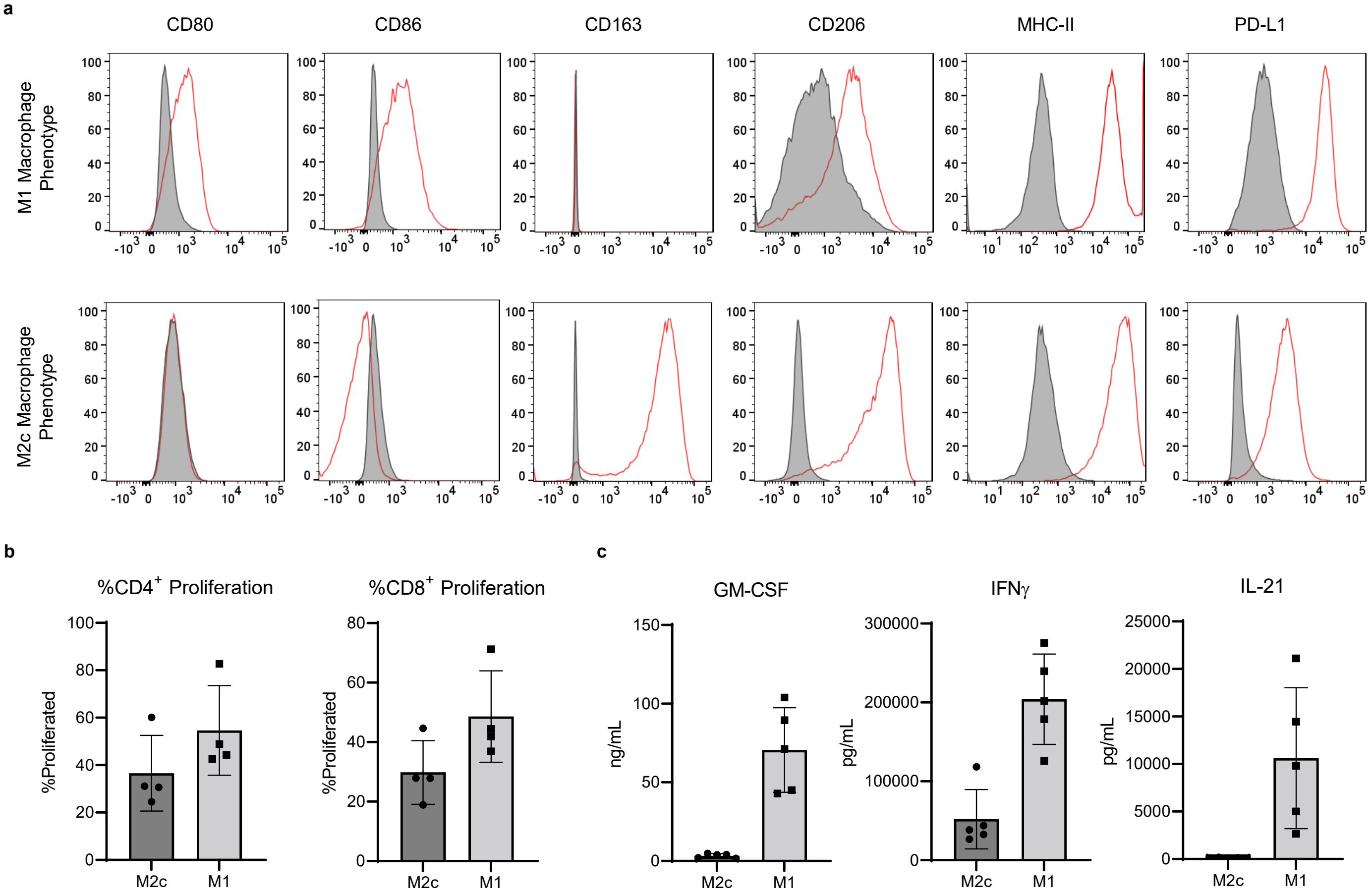

Supplementary Fig. 5 | Phenotype and function of human *in vitro*-derived macrophages. a, In the histogram plots, primary human monocyte-derived M1 macrophages and M2c macrophages were stained with phenotyping antibodies (red lines) and their corresponding isotype control antibodies (solid) after blocking the Fc gamma receptors. **b**, When cultured with autologous T cells and anti-CD3 antibody, M1 macrophages (light shaded bars) were able to induce $CD4^+$ and $CD8^+$ T-cell proliferation more potently than M2c macrophages (dark shaded bars). Each symbol represents an individual donor and the bar height represents the median value for the four donors tested ± SEM. **c,** M1 macrophages were also able to induce the secretion of pro-inflammatory cytokines (GM-CSF, IFNγ and IL-21) more potently than M2c macrophages. Each symbol represents an individual donor and the bar represents the median ± SEM for the five donors tested. Source data are provided as a Source Data file.

a

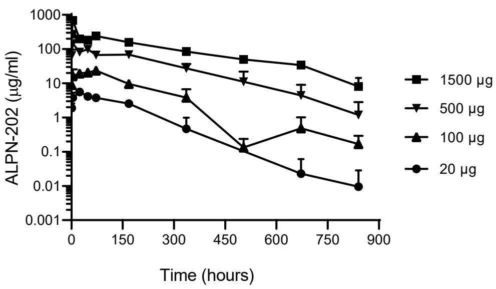

b

		ALPN-202 Dose Level (Single Intraperitoneal Injection)			
		20 µg/mouse	100 µg/mouse	500 µg/mouse	1500 µg/mouse
Parameter	Units	Estimate			
T_{max}	h	24.0	72.0	4.00	4.00
C_{max}	µg/ml	5.60	23.4	145	691
$AUC_{0-\infty}$	h*µg/ml	930	4470	25600	81100
$t_{1/2}$	H	118	NE	112	156

Supplementary Fig. 6 | Dose-proportional increase in serum concentrations of ALPN-202 following a single IP injection in MC38/hPD-L1 tumor-bearing mice. **a**, Serum concentrations of ALPN-202 versus time profiles in mice bearing human PD-L1-expressing MC38 tumors following a single IP injection of a dose range of ALPN-202. **b,** Pharmacokinetic analysis showed that serum ALPN-202 exposure increased with increasing dose. Data points shown are the mean and standard deviation of serum samples drawn from three animals per timepoint. T_{max} = time after dosing at which the maximum observed concentration was observed; C_{max} = maximum observed concentration measured after dosing; $AUC_{0-\infty}$ = area under the concentration-time curve from time 0 to extrapolated time infinity; $t_{1/2}$ = terminal half-life; NE = not estimated. Source data are provided as a Source Data file.

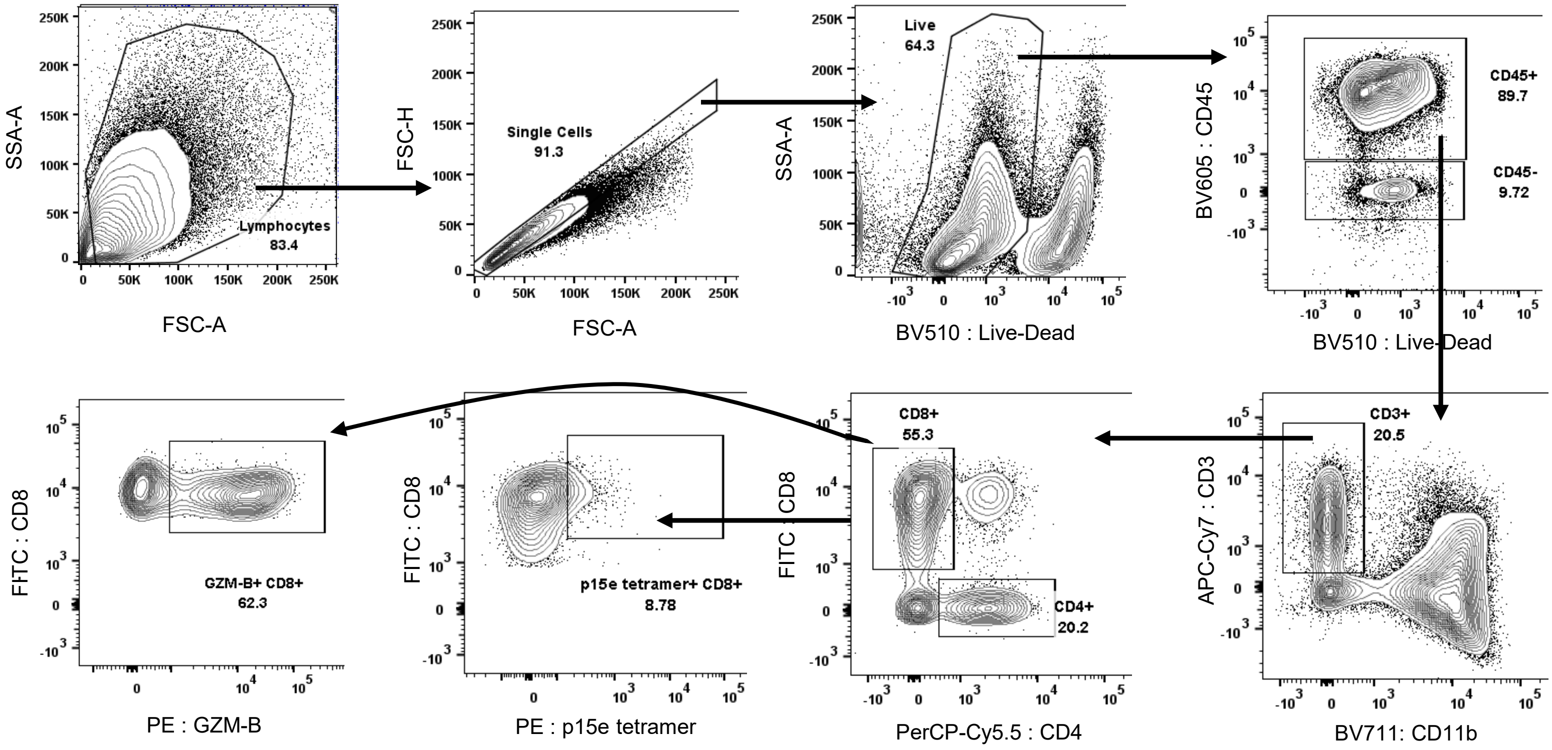

Supplementary Fig. 7 | Typical gating scheme for quantifying cell subsets in MC38/hPD-L1 tumors. In the FSC-A/SSC-A dot plot, lymphocytes were gated away from debris. A FSC-A/FSC-H gate was established along a diagonal to exclude doublet cell populations. A single cell gate was analyzed for BV510-negative cells which represent live cells that did not take up the LIVE/DEAD Aqua reagent. The live cell gate was then divided into BV605:CD45$^+$ and CD45$^-$ cells, followed by identification of APC-Cy7:CD3$^+$ cells within the CD45$^+$ gate. PerCP-Cy5.5:CD4$^+$ and FITC:CD8$^+$ T cells were gated from the CD3$^+$ population, and it was the CD8$^+$ gate that was then analyzed for PE:p15e tetramer$^+$ CD8$^+$ T cells or PE:granzyme B$^+$ (GZM-B) CD8$^+$ T cells. The mean percentages of cells in the p15e tetramer$^+$ T cell gate are displayed in **Figure 5g**. The mean percentages of parent cells in the CD3$^+$ T cell, CD8$^+$ T cell, granzyme B$^+$ T cell gates are displayed in **Figure** 6 **c-e** respectively.

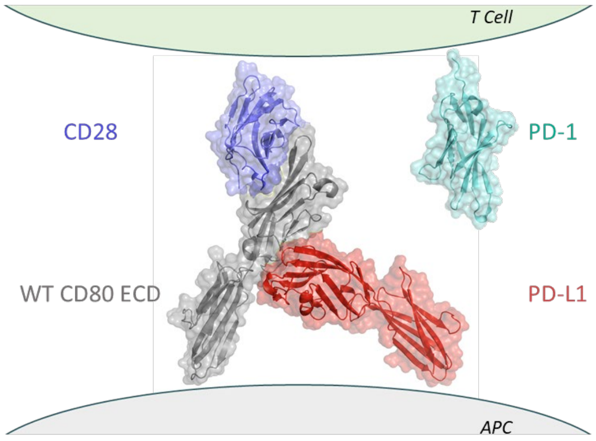

Supplementary Fig. 8 | Model of CD80 - PD-L1 *cis* interaction. Model alignments of CD80 (grey) bound to PD-L1 (red) *in cis* on the surface of an APC. CD80 bound to PD-L1 retains ability to bind CD28 (blue) *in trans* but the complex prevents PD-1 (cyan) interaction with PD-L1.

Supplementary Table 2: Crystal parameters, X-ray data processing, and refinement statistics from the ALPN-202 CD80 IgV- PD-L1 ECD structure.

	CD80 IgV – PD-L1 ECD Crystal
**Data Collection**	
Space group<br>Cell dimensions	$P2_12_12_1$
a, b, c (Å)	59.9, 122.2, 152.7
α, β, γ (º)	90, 90, 90
Resolution (Å)	95.38 - 3.15 (3.37 - 3.15)
R_{merge}(I) (%)	15.0 (172.2)
I/σI	7.6 (0.9)
Wavelength (Å)	0.9763
Completeness (%)	99.3 (98.3)
Redundancy	5.4 (4.3)
**Refinement**	
No. of observations / unique reflections	107 751 / 19 918
CC(1/2) (%)	99.6 (36.7)
R_{model}(F) (%)	24.7 (42.6)
R_{free}(F) (%)	29.1 (40.0)
No. of non-hydrogen atoms	5316
No. of water molecules	55
R.m.s. deviations from ideal geometry<br>Bond lengths (Å)	0.008
Bond angles (°)	1.1
Mean B-factor protein chain A, B, C, D ($Å^2$)	92.4, 96.5, 155.0, 115.1
Mean B-factor glycerol ($Å^2$)	78.0
Mean B-factor solvent ($Å^2$)	60.3
**Ramachandran plot quality#**	
Favored regions (%)	95.2
Allowed regions (%)	4.3
Outliers (%)	0.5

Values in parentheses are for the highest resolution shell.
#Calculated using MolProbity[59].

Supplementary Table 3. Average areas excluded in pairwise interactions between the ALPN-202 CD80 vIgD and the PD-L1 ECD calculated by AREAIMOL of the CCP4 software package.

Structure	Buried area (Å2)
CD80 (chain A)-PD-L1(chain B)	1863.5
CD80 (chain C)-PD-L1(chain D)	1917.6
Average	1890.6

Supplementary Table 4. List of antibody reagents

Flow Cytometry Reagents

Reactivity	Use	Clone	Fluor	vendor	Cat#	Dilution
human CD163	FC	GHI/61	BV421	BioLegend	333612	1:100
human CD80	FC	2D10	FITC	BioLegend	305206	1:100
human CD86	FC	FUN-1	BV510	BD Biosciences	563461	1:100
human CD206	FC	15-2	PE-Cy7	BioLegend	321124	1:100
human CD274 (PD-L1)	FC	29E.2A3	PE	BioLegend	329706	1:100
human HLA-DR, DP, DQ	FC	TU39	APC	BioLegend	361714	1:100
mouse CD3e	FC	REA606	APC-Vio 770	Miltenyi Biotec	130-117-676	1:50
mouse CD4	FC	GK1.5	PerCP-Cy5.5	BioLegend	100434	1:100
mouse CD8	FC	KT15	FITC	Thermo Fisher	MA5-16759	1:100
mouse CD11b	FC	M1/70	BV711	BioLegend	101242	1:100
mouse CD45	FC	30-F11	BV605	BioLegend	103139	1:100
mouse Granzyme B	FC	QA16A02	PE	BioLegend	372207	1:20
couse Class I p15E tetramer	FC	N/A	PE	MBL International	TB-M507-1	1:13.3

Isotype Control Antibodies

	Use	Clone	Fluor	vendor	Cat#	
mouse IgG1	FC	MOPC-21	BV421	BioLegend	400158	1:100
mouse IgG1	FC	MOPC-21	PE-Cy7	BioLegend	400126	1:100
mouse IgG2a	FC	MOPC-173	FITC	BioLegend	400208	1:100
mouse IgG2a	FC	MOPC-173	APC	BioLegend	400222	1:100
mouse IgG2b	FC	MPC-11	PE	BioLegend	400314	1:100

Functional Antibodies

Reactivity	Use	Clone	Fluor	vendor	Cat#
human PD-1	Fxn	nivolumab	None	Catalent	Special order
human PD-L1	Fxn	durvalumab	None	Catalent	Special order
human PD-L1	Fxn	atezolizumab	None	AIS	None
human CTLA-4	Fxn	ipilimumab	None	AIS	None
human CD28	Fxn	FR104-Fc	None	AIS	None
mouse CD28 (inert Fc)	In vivo	E18	None	Absolute Antibody	AB00286-3.3
mouse PD-1	In vivo	RMP1-14	None	BioXcell	BE0416
mouse CTLA-4 (inert Fc)	In vivo	9D9	None	AIS	None
mouse CTLA-4 (mIgG2b Fc)	In vivo	9D9	None	BioXcell	BP0164

FC - Flow cytometry; Fxn - Functional/blocking reagent
