# Supplementary material for: The engineered CD80 variant fusion therapeutic davoceticept combines checkpoint antagonism with conditional CD28 costimulation for anti-tumor immunity
Source: Nat Commun. 2022 Apr 4;13:1790. doi: 10.1038/s41467-022-29286-5 (PMC8980021; doi:10.1038/s41467-022-29286-5)
Supplement: Supplementary file 2 — Reporting Summary [file 41467_2022_29286_MOESM2_ESM.pdf]

nature research

Corresponding author(s): Mark Maurer

Last updated by author(s): Feb. 21, 2022

Reporting Summary

Nature Research wishes to improve the reproducibility of the work that we publish. This form provides structure for consistency and transparency in reporting. For further information on Nature Research policies, see our Editorial Policies and the Editorial Policy Checklist.

Statistics

For all statistical analyses, confirm that the following items are present in the figure legend, table legend, main text, or Methods section.

n/a	Confirmed	
☐	☒	The exact sample size (*n*) for each experimental group/condition, given as a discrete number and unit of measurement
☐	☒	A statement on whether measurements were taken from distinct samples or whether the same sample was measured repeatedly
☐	☒	The statistical test(s) used AND whether they are one- or two-sided<br>*Only common tests should be described solely by name; describe more complex techniques in the Methods section.*
☐	☒	A description of all covariates tested
☐	☒	A description of any assumptions or corrections, such as tests of normality and adjustment for multiple comparisons
☐	☒	A full description of the statistical parameters including central tendency (e.g. means) or other basic estimates (e.g. regression coefficient) AND variation (e.g. standard deviation) or associated estimates of uncertainty (e.g. confidence intervals)
☐	☒	For null hypothesis testing, the test statistic (e.g. *F*, *t*, *r*) with confidence intervals, effect sizes, degrees of freedom and *P* value noted<br>*Give P values as exact values whenever suitable.*
☒	☐	For Bayesian analysis, information on the choice of priors and Markov chain Monte Carlo settings
☒	☐	For hierarchical and complex designs, identification of the appropriate level for tests and full reporting of outcomes
☒	☐	Estimates of effect sizes (e.g. Cohen's *d*, Pearson's *r*), indicating how they were calculated

Our web collection on statistics for biologists contains articles on many of the points above.

Software and code

Policy information about availability of computer code

Data collection	Flow cytometry: Data collected using FACS Diva v8.0.1 (Beckton Dickinson, Inc.) and analyzed using FlowJo v7 and v10 software (Beckton Dickinson, Inc.).<br>RNAseq Data: Dual-index, single-read sequencing of pooled libraries was carried out on a HiSeq2500 sequencer (Illumina) with 58-base reads, using HiSeq v4 Cluster and SBS kits (Illumina) with a target depth of 5 million reads per sample.
Data analysis	Raw structural data were processed using XDS v2010 (Kabsch et al. 2010[51]) and merged using AIMLESS (Evans et al. 2006[52]).<br>The structure was determined by molecular replacement using the Molrep software (Vagin & Teplyakov, 1997[53]). Published CD80 (PDB: 1DR9 Ikemizu et.al., 2000[33]) and PD-L1 (PDB: 5JDR Zhang et.al., 2017[54]) structures were used as initial models. Two complexes of ALPN-202 and PD-L1 were found in the asymmetric unit. The structure was refined using Refmac5 v5.8.0158 (Murshudov et al., 2011[55]) and Buster v1.1.7 (Global Phasing Ltd.), and model building was carried out in Coot (Emsley P. et al, 2010[56]). Electron density map sharpening was applied in Refmac5 due to the relatively high overall temperature factors of the atoms in the crystals (the calculated "B value for simple map sharpening" was 16.4 Å2). The structure of the CD80 vIgD in complex with PD-L1 was refined to 3.15 Å. Ramachandran plot quality was calculated using a Molprobity server v4.02b-467 (Chen et al. 2010[57]) and the average area covered by the binding interactions was calculated using AREAIMOL in CCP4 software package. The binding surface and contact residues within 4 Å between the CD80 vIgD and the PD-L1 ECD were determined by using CONTACT in CCP4. AIMLESS, Molrep, Coot, AREAIMOL, and CONTACT are all part of the CCP4 Software Suite v7.0.044. Additional structural modeling was performed using The PyMol Molecular Graphics System Version 2.3.3 (Schrödinger, LLC).<br><br>RNAseq Data: Raw base calls were processed to FASTQs on BaseSpace (Illumina), and a base call quality-trimming step was applied to remove low-confidence base calls from the ends of reads. The FASTQs were aligned to the mouse reference genome, using STAR v.2.4.2a and gene counts were generated using htseq-count. QC and metrics analysis was performed using the Picard family of tools (v1.134).<br>TPMs were calculated using the gene lengths from NCBIM37 as downloaded via BioMart (ensembl.org) for the May 2012 version. The counts for each gene were divided by the length of the gene and multiplied by 1000. Then all the values were divided by the number of million reads in that sample. Evaluation of differential gene expression was performed using the R-based Needle Genomics Expression Atlas (Needle

Genomics LLC, Seattle, WA). Gene signature analysis was performed by identifying the 200 most differentially regulated genes comparing ALPN-202 with Fc-control treated tumors using false discovery rate (FDR) and focusing on those genes upregulated more than two-fold, which yielded a list of 124 genes. These 124 genes were identified using the Needle Genomics Expression Atlas and pathway analysis with this gene set was carried out using Enrichr Pathway Analysis [https://maayanlab.cloud/Enrichr/]. Population analysis

Serum concentrations of ALPN-202 were determined by an ELISA developed at AIS and pharmacokinetic parameters estimated using Phoenix WinNonlin v6.4 software (Certara)

Surface Plasmon Resonance: ALPN-202 affinity was measured on a Biacore 3000 (GE). Data were aligned, double referenced, and fit using Scrubber v2.0 software (BioLogic Software Pty, Ltd.).

Statistical analyses for all in vitro and in vivo experiments were conducted using GraphPad Prism v8.

For manuscripts utilizing custom algorithms or software that are central to the research but not yet described in published literature, software must be made available to editors and reviewers. We strongly encourage code deposition in a community repository (e.g. GitHub). See the Nature Research guidelines for submitting code & software for further information.

Data

Policy information about availability of data

All manuscripts must include a data availability statement. This statement should provide the following information, where applicable:

- Accession codes, unique identifiers, or web links for publicly available datasets
- A list of figures that have associated raw data
- A description of any restrictions on data availability

Structure coordinates of the ALPN-202 CD80 vIgD:PD-L1 ECD complex have been deposited in the worldwide Protein Data Bank (wwPDB; http://www.wwpdb/org) with accession number 7TPS [https://www.rcsb.org/structure/7tps]. RNAseq data is available at the Gene Expression Omnibus (GEO; https://www.ncbi.nlm.nih.gov/geo/) with accession# GSE161244 [https://www.ncbi.nlm.nih.gov/geo/query/acc.cgi?acc=GSE161244]. Source data for Figures 1-7 and Supplemental Figures 1-6 are available in the associated Source Data file.

Additional References Cited in this manuscript:

PDB: 1DR9 - [https://www.rcsb.org/structure/1dr9]

PDB 5JDR - [https://www.rcsb.org/structure/5jdr]

PDB 4Z18 - [https://www.rcsb.org/structure/4z18]

PDB 1I8L - [https://www.rcsb.org/structure/1i8l]

PDB 1YJD - [https://www.rcsb.org/structure/1yjd]

PDB 4ZQK - [https://www.rcsb.org/structure/4zqk]

Field-specific reporting

Please select the one below that is the best fit for your research. If you are not sure, read the appropriate sections before making your selection.

☒ Life sciences ☐ Behavioural & social sciences ☐ Ecological, evolutionary & environmental sciences

For a reference copy of the document with all sections, see nature.com/documents/nr-reporting-summary-flat.pdf

Life sciences study design

All studies must disclose on these points even when the disclosure is negative.

Sample size	During the development and evaluation of therapeutics in mouse tumor models (including the human PD-L1 transduced MC38, B16-F10, and SCC152 models in this paper), we conducted power calculations to estimate minimum sample sizes that are appropriate for determining statistically significant differences in tumor volumes over time (power = 0.80; alpha = 0.05). The results of those calculations (9-10 animals per group for tumor studies) are used for all studies in which the same model and Fc control treatment are used. For in vitro studies, at least two technical replicates were conducted for each reported experiment in Figures 1,2, and 4, and Supplementary Figures 1-5). Experiments using primary human cells (Figures 2 and 4, and Supplementary Figures 2, 3 and 5) were conducted with 3-5 donors to account for donor variability.
Data exclusions	No data were excluded from these studies.
Replication	The majority of the experiments were conducted two to three times and data shown is representative of the data. The in vivo/ex vivo studies depicted in Figure 6 were only conducted one time due to the resource constraints.
Randomization	For in vivo tumor studies, when the average tumor volume of the entire population reached 54-104 mm3, animals were staged in a random manner into groups to produce roughly equivalent mean tumor volumes per group prior to administration of test articles.
Blinding	While not officially blinded, tumor volumes were measured by caliper in a predetermined, consistent manner by individuals with no/limited knowledge of the function of the test articles.

Reporting for specific materials, systems and methods

We require information from authors about some types of materials, experimental systems and methods used in many studies. Here, indicate whether each material, system or method listed is relevant to your study. If you are not sure if a list item applies to your research, read the appropriate section before selecting a response.

Materials & experimental systems

n/a	Involved in the study
☐	☒ Antibodies
☐	☒ Eukaryotic cell lines
☒	☐ Palaeontology and archaeology
☐	☒ Animals and other organisms
☐	☒ Human research participants
☒	☐ Clinical data
☒	☐ Dual use research of concern

Methods

n/a	Involved in the study
☒	☐ ChIP-seq
☐	☒ Flow cytometry
☒	☐ MRI-based neuroimaging

Antibodies

Antibodies used	See Supplementary Table 4 for clones, fluorophores, vendors, catalog numbers, and dilutions used.
Validation	All commercial antibodies used for flow cytometry (Supplementary Table 4) have been thoroughly validated by the vendors (see validation statements below). Anti-PD-L1 antibody (durvalumab) and anti-PD-1 antibody (nivolumab) were purchased from Catalent and tested for binding to PD-L1+ or PD-1+ cells respectively by flow cytometry. Anti-PD-L1 (atezolizumab), anti-CTLA-4 (ipilimumab) and anti-human CD28 (FR104-Fc) were produced at AIS and tested by flow cytometry for binding to PD-L1, CTLA-4 and CD28 respectively. Anti-mouse CD28 (inert Fc), anti-mouse PD-1 (clone RMP1-14, rat IgG2a), and anti-mouse CTLA-4 (clone 9D9, mIgG2b) were commercially sourced and thoroughly validated by the vendors and in the scientific literature.<br><br>Validation statements from each vendor:<br>BioLegend - Specificity testing of 1-3 target cell types with either single- or multi-color analysis (including positive and negative cell types). Once specificity is confirmed, each new lot must perform with similar intensity to the in-date reference lot. Brightness (MFI) is evaluated from both positive and negative populations. Each lot product is validated by QC testing with a series of titration dilutions<br>Miltenyi Biotec - All our antibodies are rigorously tested and validated before release. In the application section on the product page, you can find examples of typical performance data. In addition, we provide extended validation data highlighting details of antibody performance, specificity, and fixation compatibility.<br>BD Biosciences - The specificity (of each antibody) is confirmed using multiple methodologies that may include a combination of flow cytometry, immunofluorescence, immunohistochemistry or western blot to test staining on a combination of primary cells, cell lines, or transfectant models. All flow cytometry reagents are titrated on the relevant positive or negative cells.<br>Thermo Fisher - Thermo Fisher Scientific is committed to adopting validation standards for our Invitrogen antibody portfolio that are tested for both target specificity and functional application. By supporting the International Working Group for Antibody Validation (IWGAV), and adopting their recommendations, we're doing our part to ensure reproducibility and proper functionality in the scientific community<br>BioXcell - Binding validation is performed for each lot for all applicable InVivoPlus™ products.<br>Absolute Antibody - No formal antibody validation statement. The anti-mCD28 (clone E18; mouse antibody) product sheet references the source and specificity of the E18 antibody in PMID: 18780833. Antibody binding to and blocking of mouse CD28 was confirmed by the authors of this manuscript.

Eukaryotic cell lines

Policy information about cell lines

Cell line source(s)	GloResponse™ IL-2-luc2P Jurkat cells were purchased from Promega(#J129A). K-562 cells were purchased from ATCC (#CCL-243) and virally transduced to stably express transmembrane anti-CD3 single chain Fv (scFv) and/or wild type PD-L1. B16-F10 cells were purchased from ATCC (#CRL-6475). SCC152 cells were purchased from ATCC (#CRL-3240). MC38 cells were a kind gift from Mark Selby who in turn received them from the laboratory of Dr James Allison (original commercial source is unknown). ExpiCHO-S™ xpi293™ cells were purchased from Thermo Fisher Scientific and and virally transduced to express human CTLA-4, CD28, or PD-L1. Expi293™ cells were purchased from ThermoFisher Scientific and used to produce proteins or were transiently transfected to express mouse CTLA-4, CD28, or PD-L1.
Authentication	Cell lines used were not authenticated
Mycoplasma contamination	Cell lines were not tested for mycoplasma contamination during the time range of these studies.
Commonly misidentified lines<br>(See ICLAC register)	No commonly misidentified cell lines were used

Animals and other organisms

Policy information about studies involving animals; ARRIVE guidelines recommended for reporting animal research

Laboratory animals	Female C57BL/6NJ mice (8 – 10 weeks old at study start) and female NSG (NOD-scid IL2Rgammanull) mice (aged 7-8 weeks) were purchased from The Jackson Laboratory and housed under specific pathogen-free conditions at the Association for Assessment and

	Accreditation of Laboratory Animal Care-approved animal facility affiliated with Alpine Immune Sciences. All mice were housed at 24 ±2 °C on a 12 h light/12 h dark cycle and the animals had free access to food and water at all times.
Wild animals	This study did not use wild animals
Field-collected samples	This study did not use wild animals
Ethics oversight	Animal care, study approval, and protocol execution were conducted under the oversight of the IACUC affiliated with Alpine Immune Sciences.

Note that full information on the approval of the study protocol must also be provided in the manuscript.

Human research participants

Policy information about studies involving human research participants

Population characteristics	Heathly donor whole blood, LRS chambers, and purified T cells from 5+ donors were purchased from a local vendor, Bloodworks Northwest. Donor population characteristics were not included in the product documentation.
Recruitment	Bloodworks NW is a licensed blood and blood products collection and distribution agency. They draw blood from the local community and supply local hospitals as well as non-profit and for-profit research institutions.
Ethics oversight	Bloodworks NW has AABB and CLIA accreditation and is authorized by the Washington State Department of Health (RCW 70.335) to be a Blood Collecting or Distributing Establishment (Credential Number BLE.FS.60794929). Blood and blood products for research purposes are collected from healthy, consented adults under under WIRB Protocol #20151321 (Bloodworks NML DONOR_BLOOD_002).

Note that full information on the approval of the study protocol must also be provided in the manuscript.

Flow Cytometry

Plots

Confirm that:

- [x] The axis labels state the marker and fluorochrome used (e.g. CD4-FITC).
- [x] The axis scales are clearly visible. Include numbers along axes only for bottom left plot of group (a 'group' is an analysis of identical markers).
- [x] All plots are contour plots with outliers or pseudocolor plots.
- [x] A numerical value for number of cells or percentage (with statistics) is provided.

Methodology

Sample preparation	MC38 tumors were enzymatically digested at 37°C for 42 min using a Mouse Tumor Digestion Kit (Miltenyi Biotec) and a gentleMACS™ Octo Dissociator with Heaters (Miltenyi Biotec) according to the manufacturer's instructions for soft and medium tumors. For each tumor sample, 0.5 – 1E6 live cells were viability stained and Fc Blocked. Cells were then incubated for 45 min on ice with a cocktail of fluorescently-labeled anti-mouse antibodies against CD3ε, CD4, CD8α, CD11b, and CD45. In the study that included a PE-conjugated mouse class I p15E tetramer to identify antigen-specific CD8+ T cells (Fig. 5d), 10 µl tetramer (MBL International) was pre-incubated with each tumor cell suspension sample for 30 min at 25°C prior to the addition of the flow antibody cocktail. For granzyme B intracellular staining, the surface-stained cells were fixed and permeabilized using an intracellular fix/perm buffer set (BioLegend) prior to a 45 min incubation at 25°C with anti-mouse granzyme B (clone QA16A02; BioLegend). All stained samples were washed and collected onto a Becton Dickinson LSRII flow cytometer. Cell subsets were identified and quantified using FlowJo v10 software.
Instrument	Becton Dickinson LSRII flow cytometer equipped with 355nm, 404nm, 488nm, 561nm, and 638nm lasers
Software	BD FACSDiva software was used to collect data. FlowJo V.7 and V.10 software was used to analyze the data.
Cell population abundance	All gated populations were >5% of the parental population and easily visualized on the relevant scatter plots. Greater than 500,000 events were captured per samples which enabled >1000 events in each of the reported gates.
Gating strategy	Mouse TIL: In the FSC-A/SSC-A dot plot, lymphocytes were gated away from debris. This gate was analyzed by FSC-A/FSC-H to gate on cells along an established diagonal to exclude doublet cell populations. The single cell gate was analyzed for BV510-negative cells which represent live cells that did not take up the LIVE/DEAD Aqua reagent. The live cell gate was then divided into CD45+ and CD45- cells, followed by identification of CD3+ cells within the CD45+ gate. CD4+ and CD8+ T cells were gated from the CD3+ population, and it was the CD8+ gate that was then analyzed for p15e tetramer+ CD8+ T cells and granzyme B + (GZM-B) CD8+ T cells.

- [x] Tick this box to confirm that a figure exemplifying the gating strategy is provided in the Supplementary Information.
