# Supplementary material for: The engineered CD80 variant fusion therapeutic davoceticept combines checkpoint antagonism with conditional CD28 costimulation for anti-tumor immunity
Source: Nat Commun. 2022 Apr 4;13:1790. doi: 10.1038/s41467-022-29286-5 (PMC8980021; doi:10.1038/s41467-022-29286-5)
Supplement: Supplementary file 3 — Description of Additional Supplementary Files [file 41467_2022_29286_MOESM3_ESM.pdf]

Title: Supplementary Data 1:
Description: EnrichR Pathway Analysis of the 124 genes upregulated > 2X in MC38/hPD-L1 tumors treated with ALPN-202. Gene counts of the top 124 upregulated (>2x) genes from MC38/hPD-L1 tumors treated with either ALPN-202 or Fc control were analysed using the EnrichR pathway analysis tool. P-values were calculated using Fisher's exact test. The adjusted P-value was calculated using the Benjamini-Hochberg method for correction for multiple hypotheses testing. Odds Ratio was computed using a modification to Fisher's exact test to indicate a risk of deviation from the expected rank. Combined score was calculated by multiplying the ln(pvalue) by the inverse of the Odds Ratio. N=4 tumor samples per group.
